# Gasdermin‐E‐Dependent Non‐Canonical Pyroptosis Promotes Drug‐Induced Liver Failure by Promoting CPS1 deISGylation and Degradation

**DOI:** 10.1002/advs.202305715

**Published:** 2024-02-28

**Authors:** Shen‐Xi Ouyang, Jia‐Hui Zhu, Qi Cao, Jian Liu, Zhen Zhang, Yan Zhang, Jing‐Wen Wu, Si‐Jia Sun, Jiang‐Tao Fu, Yi‐Ting Chen, Jie Tong, Yi Liu, Jia‐Bao Zhang, Fu‐Ming Shen, Dong‐Jie Li, Pei Wang

**Affiliations:** ^1^ Department of Pharmacy Shanghai Tenth People's Hospital School of Medicine Tongji University Shanghai 200092 China; ^2^ The Center for Basic Research and Innovation of Medicine and Pharmacy (MOE), Department of Pharmacology School of Pharmacy Naval Medical University/Second Military Medical University Shanghai 200433 China; ^3^ Shanghai Key Laboratory for Pharmaceutical Metabolite Research Naval Medical University/Second Military Medical University Shanghai 200433 China; ^4^ National Demonstration Center for Experimental Pharmaceutical Education Naval Medical University/Second Military Medical University Shanghai 200433 China; ^5^ Department of Hepatic Surgery The Eastern Hepatobiliary Surgery Hospital Naval Medical University/Second Military Medical University Shanghai 200438 China

**Keywords:** ammonia metabolism, CPS1, gasdermin, ISG15, ISGylation, liver injury, pyroptosis

## Abstract

Drug‐induced liver injury (DILI) is a significant global health issue that poses high mortality and morbidity risks. One commonly observed cause of DILI is acetaminophen (APAP) overdose. GSDME is an effector protein that induces non‐canonical pyroptosis. In this study, the activation of GSDME, but not GSDMD, in the liver tissue of mice and patients with APAP‐DILI is reported. Knockout of GSDME, rather than GSDMD, in mice protected them from APAP‐DILI. Mice with hepatocyte‐specific rescue of GSDME reproduced APAP‐induced liver injury. Furthermore, alterations in the immune cell pools observed in APAP‐induced DILI, such as the replacement of TIM4^+^ resident Kupffer cells (KCs) by monocyte‐derived KCs, Ly6C^+^ monocyte infiltration, MerTk^+^ macrophages depletion, and neutrophil increase, reappeared in mice with hepatocyte‐specific rescue of GSDME. Mechanistically, APAP exposure led to a substantial loss of interferon‐stimulated gene 15 (ISG15), resulting in deISGylation of carbamoyl phosphate synthetase‐1 (CPS1), promoted its degradation via K48‐linked ubiquitination, causing ammonia clearance dysfunction. GSDME deletion prevented these effects. Delayed administration of dimethyl‐fumarate inhibited GSDME cleavage and alleviated ammonia accumulation, mitigating liver injury. This findings demonstrated a previously uncharacterized role of GSDME in APAP‐DILI by promoting pyroptosis and CPS1 deISGylation, suggesting that inhibiting GSDME can be a promising therapeutic option for APAP‐DILI.

## Graphical abstract

Acetaminophen (APAP) overdose is the most common cause of drug‐induced liver failure (DILI), representing a significant global health issue with high mortality and morbidity risks. This study reveals that GSDME, a non‐canonical pyroptosis effector, is triggered and activated in the liver tissue of both mice and patients with APAP‐DILI, whereas the canonical pyroptosis effector GSDMD is not involved. Notably, GSDME expression in hepatocytes, rather than myeloid cells, governs pyroptosis and ammonia clearance by regulating CPS1 deISGylation and degradation. Administration of dimethyl fumarate inhibits GSDME cleavage and mitigates ammonia accumulation, thereby alleviating liver injury. These findings suggest that inhibiting GSDME holds promise as a therapeutic option for APAP‐DILI.

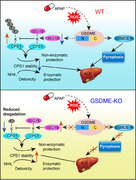

## 1 Introduction

Drug‐induced liver injury (DILI) is a significant global health issue that poses high mortality and morbidity risks.^[^ ^1^ ^]^ Acetaminophen (APAP), also known as paracetamol and widely used as an analgesic and antipyretic, is one of the most commonly observed causes of DILI.^[^ ^2^ ^]^ Despite extensive research into the mechanisms of APAP‐induced hepatotoxicity, *N*‐acetylcysteine (NAC), a reactive oxygen species (ROS) scavenger, remains the only approved clinical antidote that is effective when administered within 8–12 h after APAP ingestion.^[^ ^3^ ^]^ However, patients typically seek medical attention for APAP hepatotoxicity after the initial onset of symptoms beyond 24 h after ingestion, resulting in inevitable DILI and the need for liver transplantation. There is, therefore, an urgent need to identify new therapeutic targets for developing more effective treatments, ideally with longer dosing windows.

APAP‐induced DILI is characterized by centrilobular necrosis and high plasma transaminase levels. It is well‐accepted that APAP overdose triggers N‐acetyl‐p‐benzoquinone imine (NAPQI) formation, glutathione depletion and protein adduct formation. During this process, damage‐associated molecular patterns (DAMPs) in liver were further induced, which ultimately led to nuclear DNA damage and cell death.^[^ ^4^ ^]^ However, despite intensive efforts, the mechanisms underlying the cell death model in APAP‐induced hepatotoxicity are still not fully understood. Early studies showed that necrosis and apoptosis are the most classical forms of cell death involved in APAP‐associated hepatocyte death.^[^ ^5^ ^]^ Recently, an increasing number of novel forms of regulated cell death, such as necroptosis^[^ ^6^, ^7^ ^]^ and ferroptosis,^[^ ^8^ ^]^ have been identified and are increasingly implicated in various human pathologies. However, the roles of these less familiar modes of cell death in APAP hepatotoxicity are not well understood.

Inflammasome‐mediated pyroptosis may play a critical role in APAP‐induced hepatoxicity and inflammation. The inflammasome acts as a molecular platform, activated in response to danger‐associated signals, and triggers activation of proinflammatory caspases, leading to maturation and secretion of interleukin‐1β (IL‐1β) and IL‐18, which ultimately promotes pyroptosis, a form of lytic cell death.^[^ ^9^ ^]^ In 2015, two independent studies identified that gasdermin D (GSDMD) induces pyroptotic cell death by forming pores in plasma/mitochondrial membranes.^[^ ^10^, ^11^ ^]^ Gasdermin‐E (GSDME), another member of the gasdermin family, converts non‐inflammatory apoptosis to non‐canonical pyroptosis.^[^ ^12^, ^13^ ^]^ The gasdermin family proteins, including GSDMD, GSDME, and emerging members,^[^ ^14^, ^15^ ^]^ have now been recognized as final executors of pyroptosis. Interestingly, the GSDME pathway seems to be a backup inflammasome cascade when the canonical NLRP3/GSDMD signaling is blocked.^[^ ^16^ ^]^ The exact roles of gasdermins or pyroptosis in APAP hepatoxicity are unclear. While inhibiting inflammasome‐mediated pyroptosis has been considered a potential therapeutic option for APAP‐induced DILI,^[^ ^17^ ^]^ recent research has challenged this assumption. For instance, GSDMD deficiency failed to protect mice from high‐fat diet‐induced fatty liver, and GSDMD‐dependent pyroptosis even alleviated APAP‐related DILI.^[^ ^18^, ^19^ ^]^

This study aimed to investigate the role of GSDME‐mediated pyroptosis in APAP‐related DILI and explore its underlying mechanisms. Our findings suggest that GSDME‐mediated pyroptosis in hepatocytes, rather than in myeloid cells, drives APAP‐related DILI by mechanisms involving the promotion of deISGylation and K48‐linked ubiquitination degradation of carbamoyl phosphate synthetase‐1 (CPS‐1), a mitochondrial matrix enzyme that catalyzes the reaction of hepatic urea cycle for ammonia detoxification.

## 2 Results

### 2.1 Induced GSDME and GSDME‐Related Pyroptosis in APAP‐Related DILI

**Figure 1:**
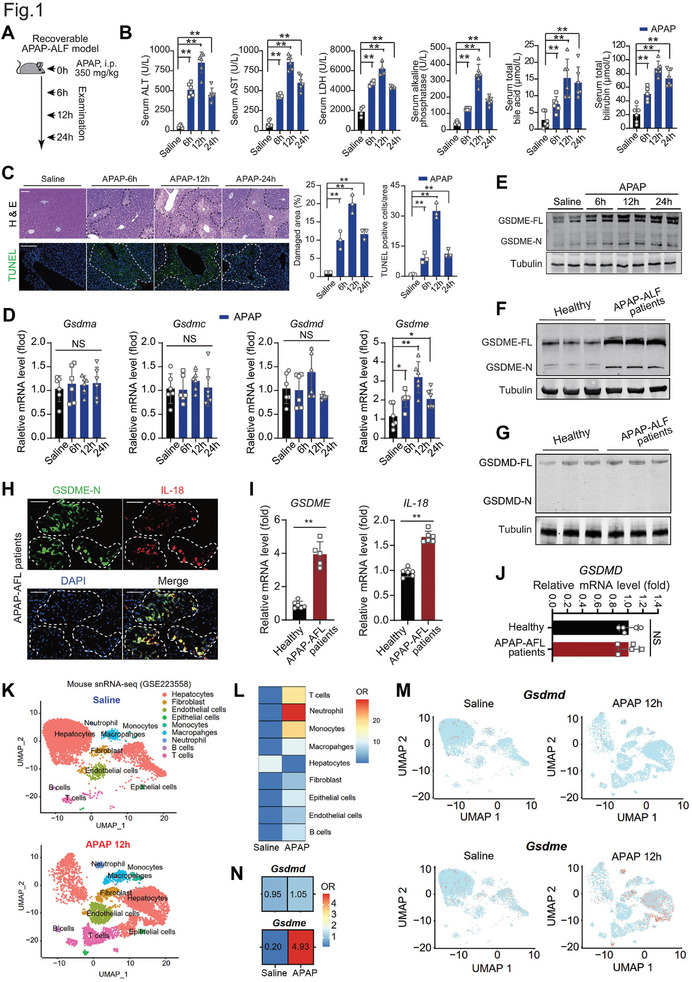
GSDME‐dependent pyroptosis is triggered in APAP‐related DILI. A) Mice were given 350 mg k^−1^ g APAP or saline (i.p.) to establish a recoverable DILI model and time points for sample harvest. B) Serum levels of alanine aminotransferase (ALT), aspartate aminotransferase (AST), lactate dehydrogenase (LDH), alkaline phosphatase (AKP), total bile acids and total bilirubin activity after APAP injection. C) Representative images and quantification analyses of H&E histological staining and TUNEL immunofluorescence staining in liver sections of mice. Scale bar = 200 µm. D) The mRNA levels of gasdermins family members (*Gsdma*, *Gsdmc*, *Gsdmd*, and *Gsdme*) in mice liver tissues. E) Immunoblotting analysis of the full length of GSDME (GSDME‐FL) and cleaved N‐terminal GSDME (GSDME‐N) in mice liver tissues. F) Immunoblotting analysis of the full length of GSDME (GSDME‐FL) and N‐terminal of GSDME (GSDME‐N, active GSDME) in liver tissue of patients with ALF. G) Immunoblotting analysis of the full length of GSDMD (GSDMD‐FL) and N‐terminal of GSDME (GSDME‐D, active GSDMD) in liver tissue of patients with ALF. Active GSDMD was not detected. H) Double‐immunofluorescent staining showing the colocalization of GSDME‐N with its downstream interleukin‐18 (IL‐18) in liver of patients with ALF. Scale bar = 100 µm. I) Intrahepatic mRNA expression of *GSDME* and *IL‐18* in liver tissue of patients with ALF. J) Intrahepatic mRNA expression of *GSDMD* in liver tissue of patients with ALF. K) A public single‐nucleus RNA sequencing‐seq (snRNA‐seq) dataset (GEO accession number: GSE223558) from liver tissue of both saline and APAP‐treated mice showing the clusters of cells with an unsupervised clustering approach using uniform manifold approximation and projection visualization (UAMP). Cells are colored by their cell type annotation. A total of 11 983 and 6443 cell nucleus were included in saline and APAP groups respectively. L) A heatmap illustrates the odds ratio (OR) of cell clusters before and after APAP stimuli. An OR > 1.5 suggests a preference for an increase in the number of cells within a specific cluster. Hierarchical clustering, based on cosine distance, is applied to the rows. M) *Gsdmd* and *Gsdme* gene expression in mice treated with saline or APAP were shown in UMAP plot. N) A heatmap illustrates the OR of *Gsdmd* and *Gsdme* mRNA expression before and after APAP stimuli. *p<0.05, **p<0.01, ***p < 0.001. NS, no significant difference, One‐Way ANOVA followed by Sidak's test. N = 6 biological replicates in A–E and I–K.

We established a recoverable DILI model in mice (APAP, i.p., 350 mg k^−1^ g) and examined the liver injury at 6, 12, and 24 h (**Figure** **1**A). Serum levels of alanine aminotransferase (ALT), aspartate aminotransferase (AST), lactate dehydrogenase (LDH), alkaline phosphatase (AKP), bile acids, and bilirubin were higher at these time points and reached a peak at 12 h post‐APAP injection (Figure 1B). Histological analysis and TUNEL assay showed a similar trend in the damaged liver area (Figure 1C). Among the four gasdermin family genes (*Gsdma, Gsdmc, Gsdmd*, and *Gsdme*), only *Gsdme* was found to be upregulated by ≈2–4 folds (Figure 1D). Immunoblotting analysis showed that the total GSDME protein level was elevated in the liver of APAP‐injected mice, while the cleavage of GSDME reached its peak at 12 h post‐APAP injection (Figure 1E). Next, we collected biopsy samples from six patients with APAP‐related DILI. Their information is illustrated in Table S1 (Supporting Information). Immunoblotting analysis demonstrated that GSDME was cleaved in the liver tissue of patients with APAP‐related DILI compared with those from healthy individuals (Figure 1F). On the contrary, GSDMD was not cleaved in the liver tissue of patients with APAP‐related DILI (Figure 1G). In the necrotic area of patient's liver tissue, the N‐terminal of GSDME (GSDME‐N), which is the active form of GSDME, is colocalized with the IL‐18 (Figure 1H). Accordingly, the gene expression of *GSDME* and *IL‐18* was significantly induced (Figure 1I), whereas *GSDMD* RNA was not upregulated (Figure 1J).

To further examine the changes in expression of *Gsdmd* and *Gsdme* following APAP stimuli, we used a public dataset of single‐nucleus RNA sequencing (snRNA‐seq, GEO accession number: GSE223558) from liver tissue of both saline and APAP‐treated mice in our analysis. After filtering out low‐quality cells from the original study, a total of 18426 cell nuclei were included in the subsequent analysis. Based on gene expression patterns, a total of 10 clusters of cells were generated (Figure 1K). The marker genes of the 10 clusters of cells are illustrated in Figure S1 (Supporting Information). The number of hepatocytes noticeably decreased, while the number of immune cells, including macrophages/monocytes, neutrophils and T cells, increased in APAP‐treated mice (Figure 1L). Using this snRNA‐seq data, we compared the transcriptome expression of *Gsdmd* and *Gsdme* in mouse liver. *Gsdmd* exhibited minimal expression in liver tissue and was unaltered after APAP treatment (Figure 1M,N). In contrast, *Gsdme* was highly expressed in liver tissue and significantly upregulated by APAP (Figure 1M,N). In another single‐cell RNA sequencing (scRNA‐seq) dataset (GEO accession number: GSE188541) in human liver organoids, we also observed an increased expression of GSDME upon APAP stimuli (Figure S2A–F, Supporting Information). These results demonstrate the induced transcription, translation, and cleavage of GSDME upon APAP stress, implying the potential critical role of pyroptosis mediated by GSDME, but not GSDMD, in APAP‐induced DILI.

### 2.2 Global Deletion of GSDME, but not GSDMD, Ameliorates APAP‐Related DILI

**Figure 2:**
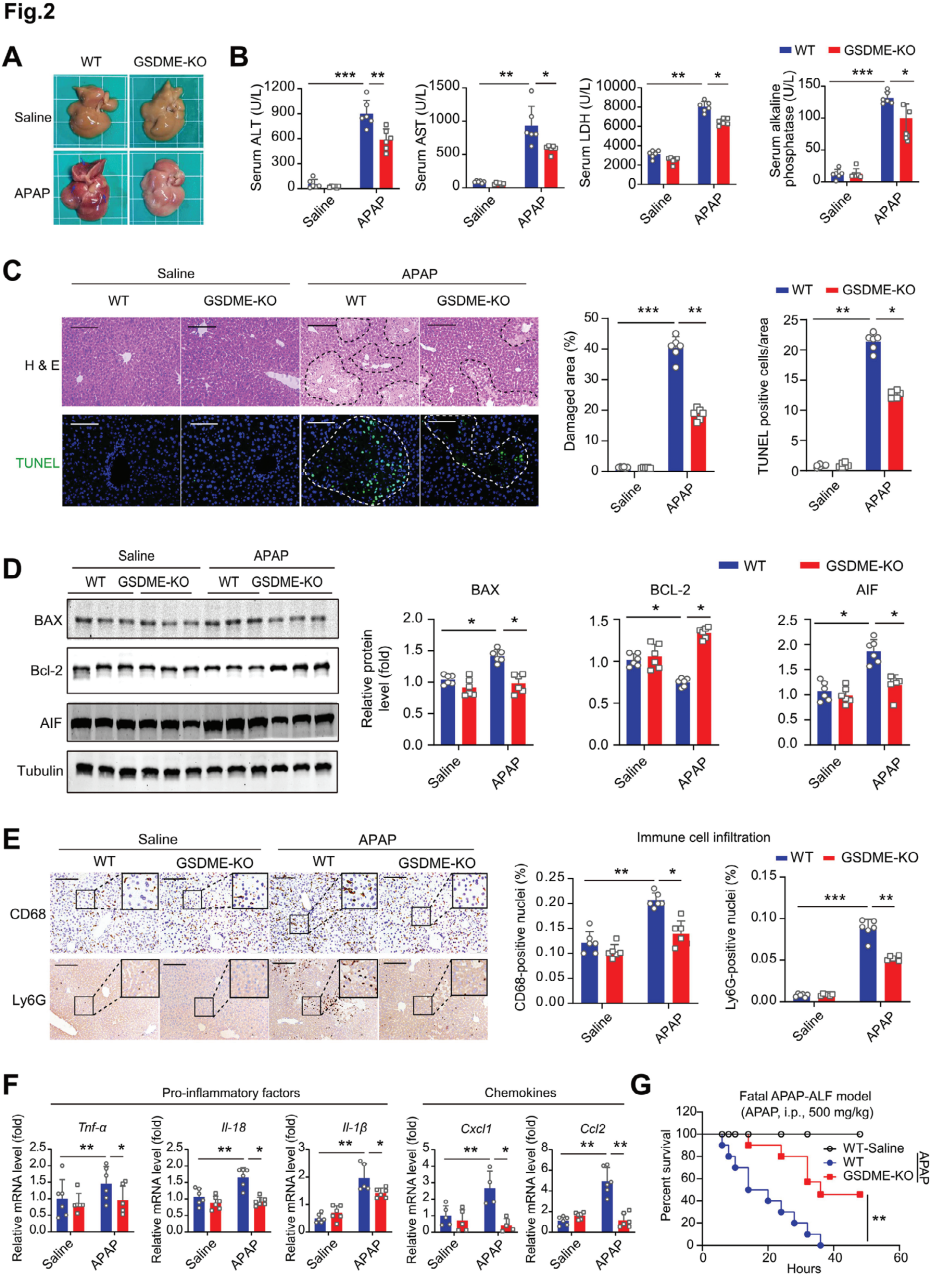
Deletion of GSDME ameliorates APAP‐induced hepatoxicity. A) Morphology of the liver from wild‐type (WT) and GSDME knockout (KO) mice upon APAP stimuli. B) Serum ALT, AST, LDH, and AKP levels in WT and KO mice treated with saline or APAP. C) Representative images and quantification analyses of H&E histological staining, and TUNEL immunofluorescence staining in liver tissue of WT and KO mice treated with saline or APAP. The dotted line content indicates the area of liver damage and TUNEL‐positive cells. Scale bar = 200 µm. D) Immunoblotting analyses of apoptosis factors including BAX, Bcl‐2, and AIF in liver tissues of WT and KO mice. E) Representative images and quantitative analysis of immunohistochemistry staining of CD68 (mouse macrophage marker) and Ly6G (neutrophil marker) in liver tissue of WT and KO mice treated with saline or APAP. Scale bar = 200 µm. F) The mRNA levels of pro‐inflammatory factors (*Tnfa*, *Il18, and Il1b*) and chemokines (*Cxcl1* and *Ccl2*) in liver tissue of WT and KO mice treated with saline or APAP. G) Survival curves of WT and KO mice treated with a lethal dose of APAP (500 mg k^−1^ g i.p.). *p<0.05, **p<0.01, One‐Way ANOVA followed by Sidak's test. N = 6 biological replicates.

To evaluate the involvement of induced GSDME in APAP‐related DILI, we generated a mouse model with global knockout (KO) of GSDME (Figure S3, Supporting Information). A recoverable DILI model was performed in wild‐type (WT) and GSDME‐KO mice. At 12 h post‐APAP treatment, bleeding points were observed on the surface of the liver in WT mice (blue arrows), which was substantially less in that of GSDME‐KO mice (**Figure** **2**A). Serum levels of ALT, AST, LDH, and AKP were increased after APAP stimuli; however, their levels in GSDME‐KO mice were lower than those in WT mice (Figure 2B). Histological and TUNEL analyses demonstrated that the necrotic area in GSDME‐KO mice was reduced (Figure 2C). APAP increased the expressions of pro‐apoptosis proteins BAX and apoptosis‐inducing factor (AIF) and decreased the expression of anti‐apoptosis factor BCL‐2, which were blunted in GSDME‐KO mice (Figure 2D). Immunohistochemistry analysis of CD68 (a macrophage/monocyte marker) and Ly6G (a neutrophil marker) demonstrated that the infiltration of MΦ/Mo and neutrophils was significantly induced in WT mice upon APAP and, to a lesser extent in GSDME‐KO mice (Figure 2E). Accordingly, the APAP‐induced gene expression of pro‐inflammatory factors (*Il‐18*, *Il‐1b*, and *Tnf‐a*) and chemokines (*Cxcl1* and *Ccl2*) was compromised by deletion of *Gsdme* (Figure 2F). Finally, in a fatal DILI model (APAP, i.p., 500 mg k^−1^ g), the deletion of Gsdme significantly extended the survival time of mice (Figure 2G). By contrast, GSDMD knockout mice did not display improved survival in such a fatal DILI model (Figure S4A, Supporting Information). There was also no alleviation in serum ALT and AST levels (Figure S4B, Supporting Information), as well as necrotic area (Figure S4C, Supporting Information) in GSDMD knockout mice. All these findings suggest that deficiency of GSDME, rather than GSDMD, mitigates APAP‐induced DILI.

### 2.3 Global Deletion of GSDME Alleviates APAP‐Induced Mitochondrial Damage and ER Stress

**Figure 3:**
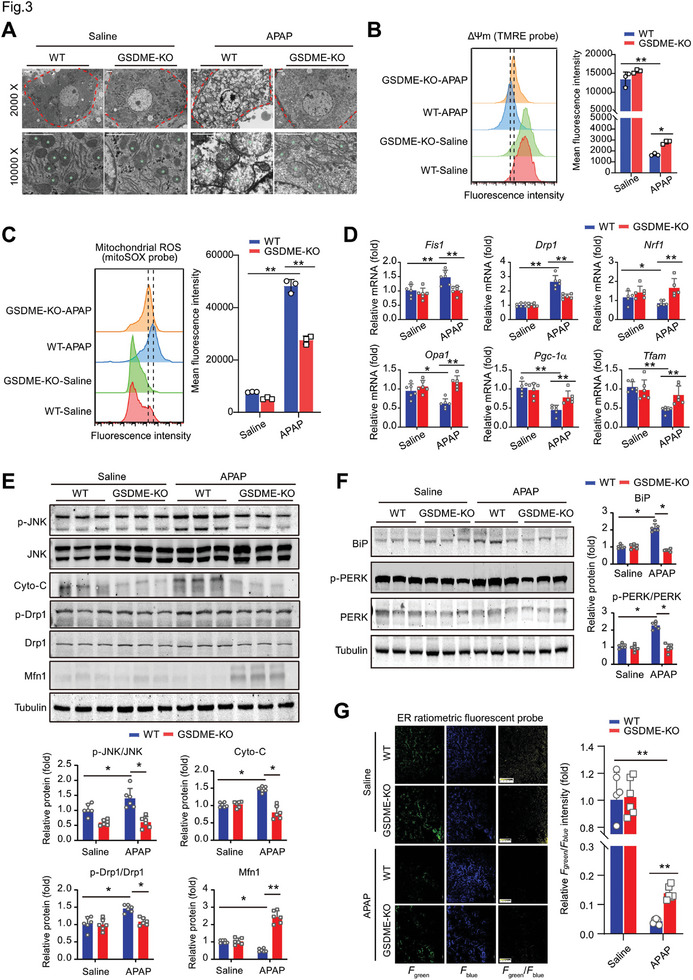
Deletion of GSDME alleviates APAP‐induced mitochondrial damage and ER stress. A) Representative electron microscopy images and analysis in mice liver tissue. Dotted line: hepatocyte. Asterisk: mitochondria. Scale bar = 10 µm. B,C) Evaluation of mitochondrial membrane potential and mitochondrial ROS production by TMRE and mitoSOX probes respectively in single‐cell solution prepared from mice liver tissue using flow cytometry. TMRE: tetramethylrhodamine, ethyl ester. D) The mRNA levels of mitochondrial fission genes (*Fis1* and *Drp1*) and mitochondrial biogenesis/functional genes (*Nrf1, Opa1, Pgc1α*, and *Tfam*) in mice liver tissue. E) Immunoblotting analyses of JNK signaling, cytochrome C, Drp‐1 phosphorylation, and Mfn1 in mice liver tissue. F) Immunoblotting analyses of ER stress markers BiP and phosphorylated protein kinase R‐like ER kinase (p‐PERK) in mice liver tissue. G) Representative images and quantification analyses of ER‐targeting ratiometric immunofluorescence staining in mice liver tissue. The ratio of fluorescence intensity in the green channel to that in the blue channel was calculated using Image J, and then the quantitative analysis was performed. Scale bar = 100 µm. *p<0.05, **p<0.01, One‐Way ANOVA followed by Sidak's test. N = 6 biological replicates.

We next evaluated mitochondrial damage and ER stress in the DILI model. Electron microscopy revealed that APAP treatment caused significant mitochondrial cavitation, swelling, rupture, and nucleolar aggregation in the livers of WT mice, which were alleviated in GSDME‐KO mice (**Figure** **3**A). Flow cytometry with TMRE probe showed a significant loss of mitochondrial membrane potential (ΔΨm) in liver cells of WT mice upon APAP, which was partially rescued in GSDME‐KO mice (Figure 3B). The mtROS content was increased by APAP in in liver cells of WT mice and, to a lesser extent, in those of GSDME‐KO mice (Figure 3C). APAP challenge increased the expression of mitochondrial fission genes (*Fis1* and *Drp1*) and reduced the expression of mitochondrial biogenesis/functional genes (*Nrf1, Opa1, Pgc1α*, and *Tfam*) in the liver tissue of WT mice. These effects were blunted in GSDME‐KO mice (Figure 3D). Immunoblotting analysis revealed that *Gsdme* deficiency prevented APAP‐induced JNK signaling activation, cytochrome C release, Drp‐1 phosphorylation, and Mfn1 loss (Figure 3E). BiP and phosphorylated protein kinase R‐like ER kinase (p‐PERK), two markers of ER stress, were induced by APAP in liver tissue but reduced by *Gsdme* deficiency (Figure 3F). Using a real‐time ER‐targeting ratiometric fluorescent probe to assess ER stress further,^[^ ^20^ ^]^ it was found that ER stress was significantly stimulated by APAP treatment in liver tissue and partially blocked by Gsdme deletion (Figure 3G). These findings suggest that GSDME deficiency alleviates hepatic mitochondrial damage and ER stress in DILI.

### 2.4 GSDME in Hepatocytes, rather than Myeloid Cells, Mediates the APAP‐Related DILI

**Figure 4:**
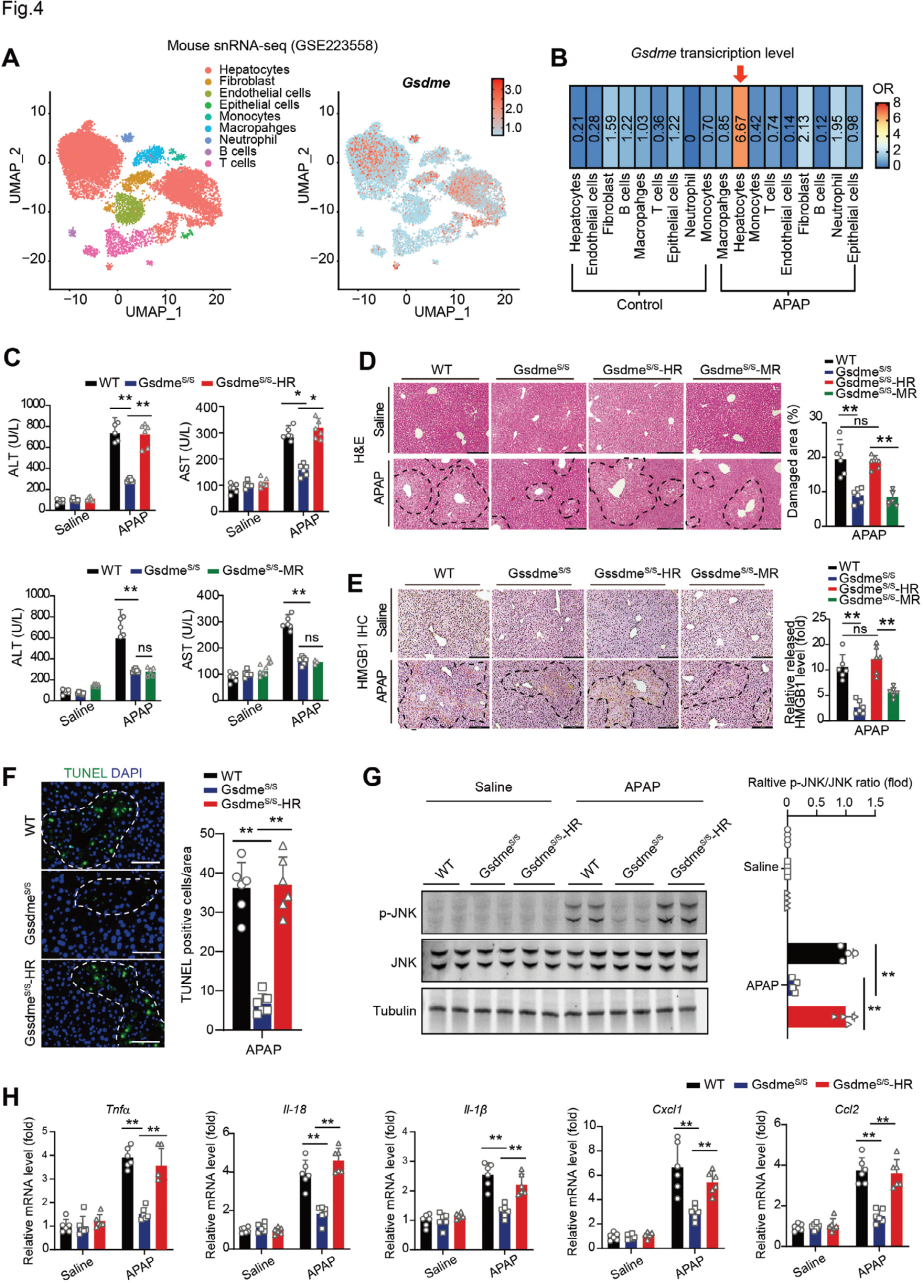
GSDME in hepatocytes, rather than myeloid cells, mediates the APAP‐induced hepatoxicity. A) UMAP dimensionality reduction analysis reveals the *Gsdme*‐expressed cells are mainly hepatocytes in mouse liver. B) Heatmap showing the distribution of *Gsdme* in each tissue before and post APAP stimuli. Hierarchical clustering based on cosine distance is applied for rows. The naming, numbering, and colors of the cell clusters are in accordance with (A). C) Serum ALT and AST levels in mice treated with saline or APAP. WT, *Gsdme* ^S/S^, *Gsdme* ^S/S^‐HR (hepatocyte rescue of GSDME), and *Gsdme* ^S/S^‐MR (myeloid cell rescue of GSDME) mice were used. D,E) Representative images and quantification analyses of H&E staining D) and HMGB1 immunohistochemistry E) in WT, *Gsdme* ^S/S^, *Gsdme* ^S/S^‐HR (hepatocyte rescue of GSDME) and *Gsdme* ^S/S^‐MR (myeloid cell rescue of GSDME) mice treated with saline or APAP. Scale bar = 200 µm. F) Representative images and quantitative analysis of TUNEL immunofluorescence staining in liver tissues of mice treated with saline or APAP. The dotted line content indicates the positive area. Scale bar = 100 µm. G) Immunoblotting analyses of JNK activation in liver tissues of mice treated with saline or APAP. (H) The mRNA levels of pro‐inflammatory factors (*Tnfa*, *Il18, and Il1b*) and chemokines (*Cxcl1* and *Ccl2*) in liver tissue of mice treated with saline or APAP. *p<0.05, **p<0.01, One‐Way ANOVA followed by Sidak's test. N = 3‐6 biological replicates.

Analysis of snRNA‐seq data (GSE223558) from liver tissue of APAP‐treated mice showed that *Gsdme* was predominantly expressed in hepatocytes (**Figure** **4**A). Notably, the mRNA level of *Gsdme* was induced by ≈10‐fold in hepatocytes following APAP stimuli (red arrow, Figure 4B). To further elucidate the cell type responsible for GSDME‐mediated pyroptosis in response to APAP‐induced hepatoxicity, a mouse model carrying a transcriptional STOP element (3×SV40‐PolyA) flanked by *loxP* (*loxP‐Stop‐loxP*)^[^ ^21^ ^]^ upstream of the ATG start codon of *Gsdme* gene was generated (Figure S5A,B, Supporting Information). The *Gsdme* ^Stop/Stop^ (*Gsdme* ^S/S^) mouse was crossed with *Alb‐Cre* or *Lysm‐Cre* mouse to produce *Gsdme* ^Stop/Stop^;*Alb*‐Cre mouse (hepatocytes rescue of GSDME, referred as *Gsdme* ^S/S^‐HR) or *Gsdme* ^Stop/Stop^;*Lysm*‐Cre mouse (myeloid cells rescue of GSDME, referred as *Gsdme* ^S/S^‐MR, Figure S5C, Supporting Information) with tissue‐specific rescue of GSDME in primary hepatocyte and myeloid cell (Figure S5D,E, Supporting Information). WT, *Gsdme* ^S/S^, *Gsdme* ^S/S^‐HR, and *Gsdme* ^S/S^‐MR mice were challenged with APAP. *Gsdme* ^S/S^ mice were resistant to APAP‐induced liver damage. Compared with WT mice, *Gsdme* ^S/S^ displayed reduced serum ALT and AST activities upon APAP (Figure 4C). The *Gsdme* ^S/S^‐HR mice exhibited comparable serum ALT and AST activities to those observed in WT mice, whereas *Gsdme* ^S/S^‐MR mice did not (Figure 4C). Similarly, compared with WT mice, the liver necrosis area and HMGB1‐positive area were reduced in *Gsdme* ^S/S^ and *Gsdme* ^S/S^‐MR mice, but not *Gsdme* ^S/S^‐HR (Figure 4D,E). Furthermore, the *Gsdme* ^S/S^‐HR mice had comparable TUNEL^+^ area (cell death), JNK activation, and pro‐inflammatory factors/chemokines as observed in WT mice (Figure 4F–H). In contrast, *Gsdme* ^S/S^‐MR mice were still resistant to APAP‐induced liver damage (Figure S6A–C, Supporting Information). These findings suggest that restoring GSDME expression in hepatocytes, but not in myeloid cells, is sufficient to reproduce APAP‐induced hepatoxicity.

### 2.5 Hepatocyte GSDME Controls the Infiltration and Molecular Status of Macrophages/Monocytes (MΦ/Mo) and Neutrophils in Response to APAP Stimuli

**Figure 5:**
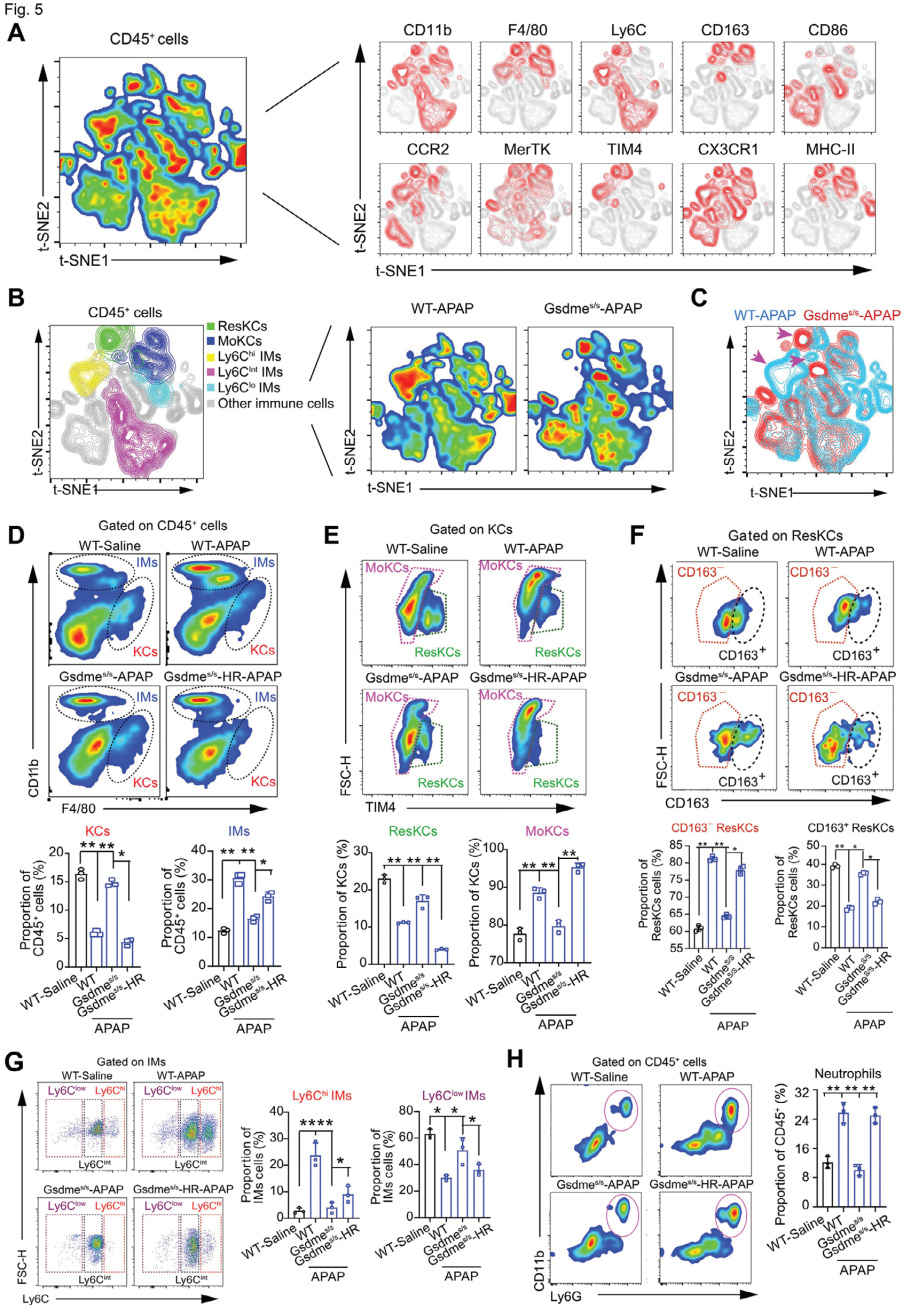
Multiplexed flow cytometry reveals the regulatory action of hepatocyte GSDME on infiltration and activation of macrophages/monocytes and neutrophils in response to APAP stimuli. A) High‐dimensional analysis of multiplexed flow cytometry using an unbiased nonlinear dimensionality reduction algorithm (t‐distributed stochastic neighbor embedding, t‐SNE) to identify clustering of subpopulations in hepatic CD45^+^ leukocyte cells. The live CD45^+^ leukocyte cells were gated with macrophages/monocytes‐related markers (CD11b, F4/80, Ly6C, CD163, CD86, CCR2, MerTK, TIM4, CX3CR1 and MHC‐II). The multiplexed flow cytometry results were concatenated, transformed, and plotted in 2D t‐SNE plots using R software. The contour plots show the relative expression of the indicated markers of myeloid cells. B) Cluster identity confirmed by manual gating (see Figure S7, Supporting Information) and t‐SNE density‐plots of CD45^+^ cells from APAP‐treated WT and Gsdme^S/S^ mice respectively. Blue, lowest frequency; red, highest frequency. C) Comparison of t‐SNE dimensionality reduction and embedding of CD45^+^ cells pooled from WT (blue) and Gsdme^S/S^ (red) mice. Pink arrow indicates the differences in clusters between WT and Gsdme^S/S^ mice upon APAP stimuli. D) Representative flow cytometry plots and quantification analyses on the proportions of Kupffer cells (KCs, F4/80^hi^CD11b^int^) and infiltrating monocytes (IMs, F4/80^int^CD11b^hi^) in CD45^+^ cells from liver tissues of WT, WT‐APAP, Gsdme^S/S^‐APAP and Gsdme^S/S^‐HR‐APAP mice. E) Representative flow cytometry plots and quantification analyses on the proportions of embryo‐derived resident KCs (ResKCs, F4/80^hi^CD11b^int^TIM4^hi^) and monocyte‐derived KCs (MoKCs, F4/80^hi^CD11b^int^TIM4^lo^) in KCs of liver tissues of WT, WT‐APAP, Gsdme^S/S^‐APAP and Gsdme^S/S^‐HR‐APAP mice. F) Representative FACS plots and quantification analyses on the proportion of M2‐like ResKCs (CD163^+^) in ResKCs (TIM4^hi^F4/80^hi^CD11b^int^) in liver tissues of WT, Gsdme^s/s^, and Gsdme^s/s^‐HR mice treated with saline or APAP. G) Representative flow cytometry plots and quantification analyses of three subclusters of IMs (Ly6C^hi^ IMs, CD11b^hi^F4/80^int^Ly6C^hi^; Ly6C^int^ IMs, CD11b^hi^F4/80^int^Ly6C^int^; Ly6C^lo^ IMs, CD11b^hi^F4/80^int^Ly6C^lo^) in IMs of liver tissues of WT, WT‐APAP, Gsdme^S/S^‐APAP and Gsdme^S/S^‐HR‐APAP mice. H) Representative flow cytometry plots and quantification analyses of neutrophils (CD45^+^CD11b^+^Ly6G^+^) in liver tissues of WT, WT‐APAP, Gsdme^S/S^‐APAP and Gsdme^S/S^‐HR‐APAP mice. *p<0.05, **p<0.01, One‐Way ANOVA followed by Sidak's test. N = 3‐6 biological replicates.

Hepatic MΦ/Mo populations, primarily composed of Kupffer cells (KCs) and infiltrating monocytes (IMs), play a crucial role in both inducing and resolving APAP‐induced hepatoxicity. KCs can be further categorized as embryo‐derived resident KCs (ResKCs) and monocyte‐derived KCs (MoKCs), which resolve and fuel hepatic inflammation respectively.^[^ ^22^ ^]^ Following injury, the self‐renewal of ResKCs is impaired, leading to chronic replacement by MoKCs.^[^ ^23^ ^]^ We next evaluated the influence of GSDME‐dependent pyroptosis on hepatic MΦ/Mo phenotypes. CD45^+^ single cells isolated from the mouse liver were stained with antibodies against CD11b, F4/80, Ly6C, CD163, CD86, CCR2, MerTK, TIM4, CX3CR1, and MHC‐II in multiplexed flow cytometry. The data were then analyzed using an unbiased non‐linear high‐dimensional analysis approach (**Figure** **5**A). With t‐distributed stochastic neighbor embedding (t‐SNE)‐guided gating, several MΦ/Mo clusters, including ResKCs (F4/80^hi^CD11b^int^TIM4^hi^), MoKCs (F4/80^hi^CD11b^int^TIM4^lo^), Ly6C^hi^ IMs (CD11b^hi^F4/80^int^Ly6C^hi^), Ly6C^int^ IMs (CD11b^hi^F4/80^int^Ly6C^int^) and Ly6C^lo^ IMs (CD11b^hi^F4/80^int^Ly6C^lo^), were identified (Figure 5B). Differences in the distributions and frequencies of ResKCs, MoKCs, and Ly6C^hi^ IMs clusters were observed between WT and *Gsdme^S/S^* mice upon APAP stimuli (pink arrow, Figure 5C).

The impact of GSDME‐dependent pyroptosis on MΦ/Mo dynamics was further evaluated using flow cytometry with manual gating (Figure S7, Supporting Information). Based on classical markers of macrophage polarization (M1‐like: CD86; M2‐like: CD163), *Gsdme* ^S/S^ mice showed a reduced or an increased frequency of M1‐ and M2‐like MΦ/Mo respectively compared with WT mice upon APAP, which were blunted in *Gsdme* ^S/S^‐HR mice (Figure S8A, Supporting Information). In WT mice, the frequency of hepatic KCs declined, while the frequency of IMs increased upon APAP; these changes were partially prevented in *Gsdme* ^S/S^ mice but reappeared in *Gsdme* ^S/S^‐HR mice (Figure 5D). Additionally, the depletion of F4/80^hi^CD11b^int^TIM4^hi^ ResKCs and induction of F4/80^hi^CD11b^int^TIM4^lo^ MoKCs in response to APAP were attenuated in *Gsdme* ^S/S^ mice but not in *Gsdme* ^S/S^‐HR mice (Figure 5E). When the ResKCs were divided into CD163^+^ and CD163^−^ ResKCs, only CD163^+^ ResKCs exhibited a similar change as ResKCs (Figure 5F). Not surprisingly, the frequencies of Ly6C^hi^ IMs (classical monocytes) and Ly6C^lo^ IMs (non‐classical monocytes) were increased and decreased respectively by APAP in WT mice, which was compromised in *Gsdme* ^S/S^ mice but reproduced in *Gsdme* ^S/S^‐HR mice (Figure 5G). Hepatic MΦ/Mo expressing MerTK efferocytosis receptor is critical for resolving inflammation in APAP‐induced hepatotoxicity.^[^ ^24^ ^]^ We observed a significant decline in the total number of MerTk^+^ MΦ/Mo upon APAP stimuli, which was blocked in *Gsdme^S/S^* mice but rescued in *Gsdme^S/S^*‐HR mice (Figure S8B, Supporting Information). When we further distinguished the MerTk^+^ KCs and IMs with MHC‐II (an anti‐inflammatory marker with antigen‐presenting activity) and Ly6C (a classically pro‐inflammatory marker), it was found that there was a decrease in MHC‐II expression in MerTk^+^ KCs and an increase in Ly6C expression in MerTk^+^ IMs, which were attenuated in *Gsdme^S/S^* mice but rescued in *Gsdme^S/S^*‐HR mice (Figure S7C–E, Supporting Information). Finally, the infiltration of neutrophils was also inhibited in *Gsdme^S/S^* mice and rescued in *Gsdme^S/S^*‐HR mice (Figure 5H). Collectively, these results indicate that GSDME in hepatocytes governs the infiltration and activation of MΦ/Mo and neutrophils in response to APAP.

### 2.6 Carbamoyl Phosphate Synthetase‐1 (CPS1) is deISGylated upon APAP in a GSDME‐Dependent Manner

**Figure 6:**
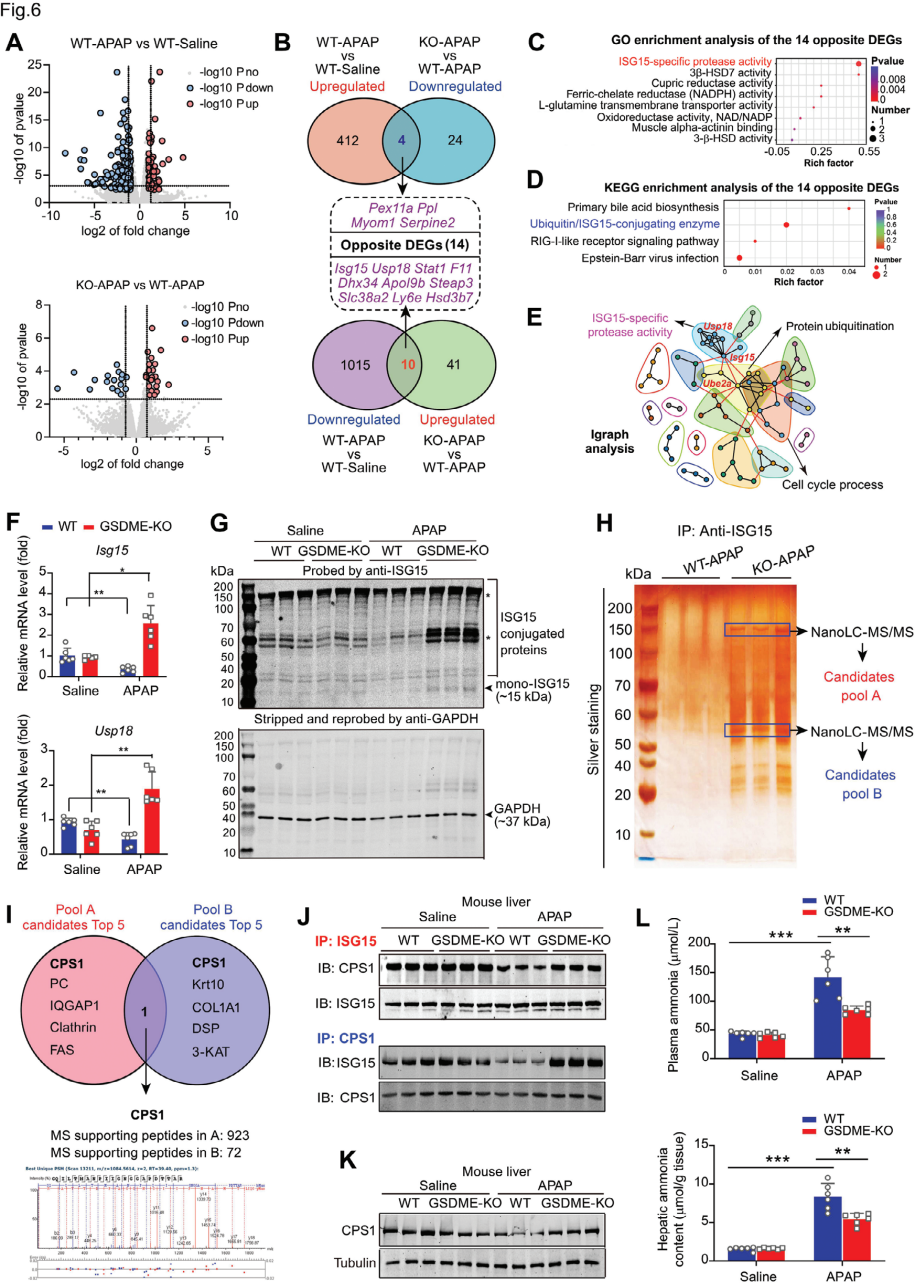
GSDME controls deISGylation of carbamoyl phosphate synthetase‐1 (CPS1) upon APAP. A) Volcano plot showing the differentially expressed genes (DEGs) altered by APAP stimuli (WT+APAP vs. WT+Saline) and GSDME knockout (KO‐APAP vs. WT‐APAP) in RNA‐seq. B) Venn plot showing the strategy for screening the opposite DEGs in the liver altered by APAP stimuli and GSDME knockout. C,D) Gene ontology (GO) and Kyoto Encyclopedia of Genes and Genomes (KEGG) analyses show the enrichment results of the 14 opposite DEGs obtained in (B). E) Igraph R package tool was applied to analyze the possible gene‐network using upregulated and downregulated genes (KO‐APAP vs. WT‐APAP). The graphical representation of a pathway/network consists of the nodes (genes) and edges (biological relationship between nodes), with characteristic symbols for the different types of molecules. Subnetworks (Neighborhoods) are colored and annotated with enriched functional categories. Gray lines: connections within a neighborhood; Red lines: connections between neighborhoods. F) The mRNA levels of *Isg15* and *Usp18* in liver tissue of WT and KO mice. G) ISGylation status in liver of WT and KO mice upon APAP. ISG15‐conjugated proteins (>40 kDa) and the mono‐ISG15 (≈15 kDa) were shown. GAPDH was used as an internal reference. H) Identification of ISG15‐interacting molecule in liver tissues of WT and KO mice treated with APAP using NanoLC‐MS/MS analysis. The ISGylated‐proteins candidates obtained in ≈150 kDa band were designed as Pool A, and the candidates obtained in the ≈50–60 kDa band were designed as Pool B. I) Identification of carbamoyl phosphate synthetase‐1 (CPS1) as the most pronounced ISGylated protein. J) ISGylation of CPS1 in liver tissues of WT and KO mice treated with saline or APAP. K) Protein expression of CPS1 in liver tissues of WT and KO mice treated with saline or APAP. L,M) Levels of ammonia in plasma (L) and liver (M) of WT and KO mice treated saline or APAP. *p<0.05, **p<0.01, ***p<0.001, One‐Way ANOVA followed by Sidak's test. N = 3‐6 biological replicates.

Next, a transcriptome RNA‐sequencing analysis was performed to explore the molecular events associated with GSDME deficiency in APAP hepatoxicity (Figure S9A, Supporting Information). The differentially expressed genes (DEGs) induced by APAP between WT or GSDME‐KO mice were identified (**Figure** **6**A; Figure S9B,C, Supporting Information). Fourteen differentially expressed genes (DEGs), including *Pex11a, Ppl, Myom1, Serpine2, lsg15, Usp18, Stat1, F11, Dhx34, Apo19b, Steap3, SIc38a2, Ly6e*, and *Hsd3b7*, were identified based on the screening criteria (Figure 6B). We used the 14 DEGs to conduct bioinformatics analyses, including Gene Ontology (GO) analysis, Kyoto Encyclopedia of Genes and Genomes (KEGG) analysis and igraph analysis. Interestingly, the three types of bioinformatics analyses showed interferon‐stimulated gene 15 (ISG15)‐specific protease activity were enriched (Figure 6C–E). This suggests that ISG15 may be a critical factor in GSDME‐dependent pyroptosis and APAP‐induced hepatoxicity.

ISG15 is an interferon (IFN)‐induced ubiquitin‐like protein that plays multifaceted roles, serving not only as a free intracellular molecule but also as a regulator in the ISG15 conjugation process (ISGylation), a type of post‐translational modification (PTM).^[^ ^25^ ^]^ Ubiquitin‐specific protease 18 (USP18) constitutes the major enzyme for the de‐ISGylation of ISG15‐conjugated proteins (Figure S9D, Supporting Information).^[^ ^25^ ^]^ We confirmed that the hepatic mRNA levels of *Isg15* and *Usp18* were downregulated upon APAP in WT mice but rescued in GSDME‐KO mice (Figure 6F). Immunoblotting showed that the mono‐ISG15 (≈15 kDa) and ISG15‐conjugated proteins (>40 kDa) were reduced in liver tissues of APAP‐treated WT mice but massively increased in GSDME‐KO mice (Figure 6G).

Next, we identified potential ISGylated proteins using immunoprecipitation and NanoLC‐MS/MS technology. Silver staining showed that *Gsdme* deletion obviously increased ISGylation in liver, and two notable bands appeared ≈150 and ≈50–60 kDa (Figure 6H). In subsequent NanoLC‐MS/MS analysis, CPS1 was identified as the top 1 candidate of ISGylated‐protein within both bands (Figure 6I). CPS1 is the rate‐limiting enzyme for the transformation of ammonia into the urea cycle in hepatocytes (Figure S9E, Supporting Information).^[^ ^26^ ^]^ We confirmed that ISGylated‐CPS1 was downregulated by APAP in WT mice but not in GSDME‐KO mice (Figure 6J). CPS1 also declined upon APAP in WT mice but not in GSDME‐KO mice (Figure 6K). Overexpression of GSDME or knockdown of ISG15 promoted CPS1 protein decline (Figure S10A,B, Supporting Information). Overexpression of GSDME also decreased the ISGylation of CPS1 (Figure S10C, Supporting Information). Both the plasma and hepatic ammonia were increased significantly after APAP in WT mice and, to a much lesser extent in GSDME‐KO mice (Figure 6L). These findings indicate that CPS1 is deISGylated upon APAP in a GSDME‐dependent manner.

### 2.7 CPS1 deISGylation Promotes its K48‐Linked Ubiquitination and Degradation upon APAP

**Figure 7:**
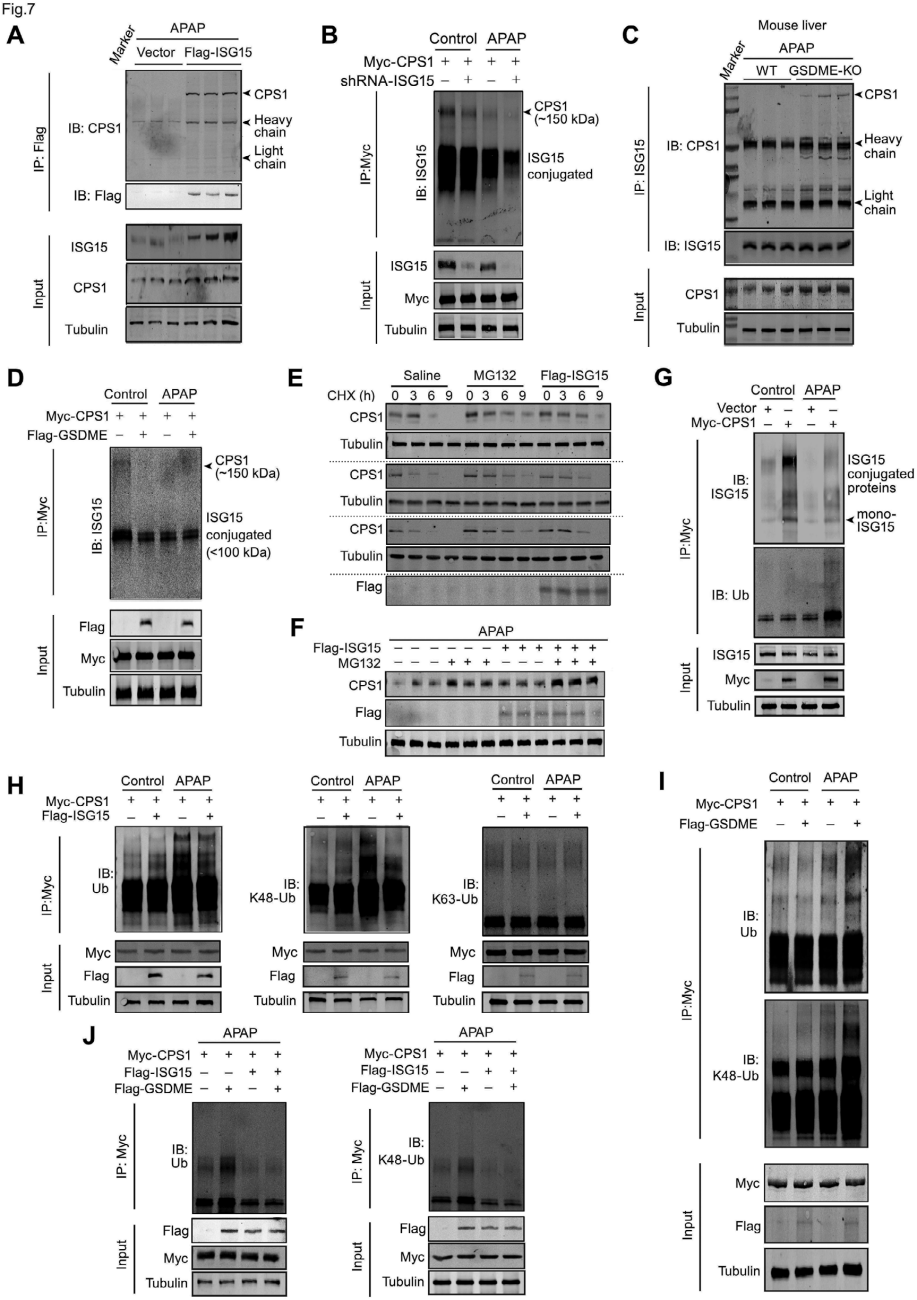
CPS1 deISGylation promotes K48‐linked ubiquitination degradation of CPS1 upon APAP‐induced hepatoxicity. A) The interaction between endogenous CPS1 and exogenous ISG15 was detected in AML12 cells. B,C) ISGylation of CPS1 in AML12 cells (B) and mouse liver (C) under knockdown of ISG15 and GSDME deficiency. D) ISGylation of CPS1 in AML12 cells overexpressed with GSDME. E) Determination of CPS1 protein stability in AML12 cells in the presence of cycloheximide (CHX), proteasome inhibitor MG132 and overexpression of ISG15. F) CPS1 protein level in AML12 cells with ISG15 overexpression, MG132, and MG132 plus ISG15 overexpression. G) ISGylation and ubiquitination of CPS1 in APAP‐treated AML12 cells. H) Total ubiquitination, K48 linked‐ubiquitination, and K63 linked‐ubiquitination of CPS1 in AML12 cells overexpressed with ISG15. I) Total ubiquitination and K48 linked ubiquitination of CPS1 in AML12 cells overexpressed with GSDME. J) Total ubiquitination and K48 linked ubiquitination of CPS1 in AML12 cells overexpressed with GSDME and ISG15. IP: immunoprecipitation. IB: immunoblotting.

CPS1 is secreted from the liver to bile or blood and exerts its protection against DILI via non‐enzymatic mechanisms.^[^ ^27^ ^]^ We further studied the molecular mechanisms underlying the modulation of GSDME on deISGylation and downregulation of CPS1. An interaction between ISG15 and endogenous CPS1, as demonstrated by co‐immunoprecipitation, was detected in AML12 hepatocytes (**Figure** **7**A). APAP reduced ISGylation of CPS1, which was further promoted by ISG15 knockdown in AML12 hepatocytes (Figure 7B). Interestingly, ISGylated‐CPS1 was rarely detected in the liver of WT mice but reappeared in the liver of KO mice (Figure 7C), indicating that deletion of *Gsdme* rescued CPS1 ISGylation. Accordingly, *Gsdme* overexpression reduced CPS1 ISGylation (Figure 7D). It is noteworthy that ISGylated‐CPS1 was detected in the low molecular weight area (<100 kDa) and the original form (≈150 kDa, Figure 7B,D), suggesting that the low‐molecular‐weight ISGylated‐CPS1 might represent degraded CPS1.

Therefore, we tested whether CPS1 was degraded via ubiquitin/proteasome pathway. Proteasome inhibitor MG132 increased CPS1 protein levels in the presence of cycloheximide (CHX), confirming that CPS1 is degraded via a ubiquitination‐dependent pathway (Figure 7E). Interestingly, ISG15 overexpression inhibited the degradation of CPS1 protein in CHX conditions (Figure 7E). Importantly, both MG132 and overexpression of ISG15 increased CPS1 protein level under APAP stimuli (Figure 7F), suggesting that the APAP‐induced CPS1 loss is mediated by deISGylation and ubiquitination/degradation. These results raised the possibility that ISGylation of CPS1 inhibits its ubiquitination and degradation. In support of this assumption, APAP caused a decline of CPS1 ISGylation and an increased CPS1 ubiquitination in hepatocytes (Figure 7G). We further analyzed which type of ubiquitination of CPS1 was regulated by its ISGylation. Overexpression of ISG15 inhibited APAP‐induced total and K48‐linked ubiquitination of CPS1 but not K63‐linked ubiquitination (Figure 7H). Accordingly, GSDME overexpression promoted CPS1 total and K48‐linked ubiquitination (Figure 7I). Simultaneous overexpression of ISG15 suppressed the enhanced total and K48‐linked ubiquitination of CPS1 induced by GSDME overexpression (Figure 7J). All these findings indicate that CPS1 deISGylation promotes its K48‐linked ubiquitination and degradation upon APAP in a GSDME‐dependent manner.

### 2.8 Accumulation of Ammonia Resulting from the deISGylation and Degradation of CPS1 Contributes to APAP‐Induced Liver Injury

In the subsequent investigation, we aimed to assess the role of ammonia accumulation in APAP‐induced liver injury. L‐ornithine phenylacetate (OP), a well‐established ammonia‐clearing agent previously reported as an effective strategy for treating hyperammonemia,^[^ ^28^, ^29^ ^]^ was administered (0.5 g kg^−1^) at 2 h post‐APAP or saline injection (Figure S11A, Supporting Information). As anticipated, OP treatment successfully reduced both serum and intrahepatic ammonia levels in both controls (saline‐treated) and APAP‐treated mice (Figure S11B, Supporting Information). Furthermore, OP treatment significantly decreased the liver necrotic area, as well as ALT and AST levels (Figure S11C,D, Supporting Information). A notable reduction in the number of TUNEL^+^ dead cells was also observed in OP‐treated mice (Figure S11E, Supporting Information).

We also explored whether interleukin‐1β (IL‐1β)/IL‐18 released by pyroptosis are major contributors to CPS1 deISGylation. Combined administration of anti‐IL‐1β and anti‐IL‐18 successfully inhibited the increases of serum ALT and AST levels (Figure S12A, Supporting Information) in APAP‐treated mice, indicating the protective effect of combined anti‐IL‐1β and anti‐IL‐18 against APAP‐induced hepatocyte injury. However, this treatment failed to block the deISGylation of CPS1 (Figure S12B, Supporting Information) and the decline in total CPS1 protein (Figure S12C, Supporting Information). These findings collectively suggest that the deISGylation and degradation of CPS1, occurring through a mechanism independent of pyroptosis‐executive molecules, lead to ammonia accumulation and subsequent liver injury.

### 2.9 Delayed Pharmacological Inhibition of GSDME Alleviates APAP‐Related DILI

**Figure 8:**
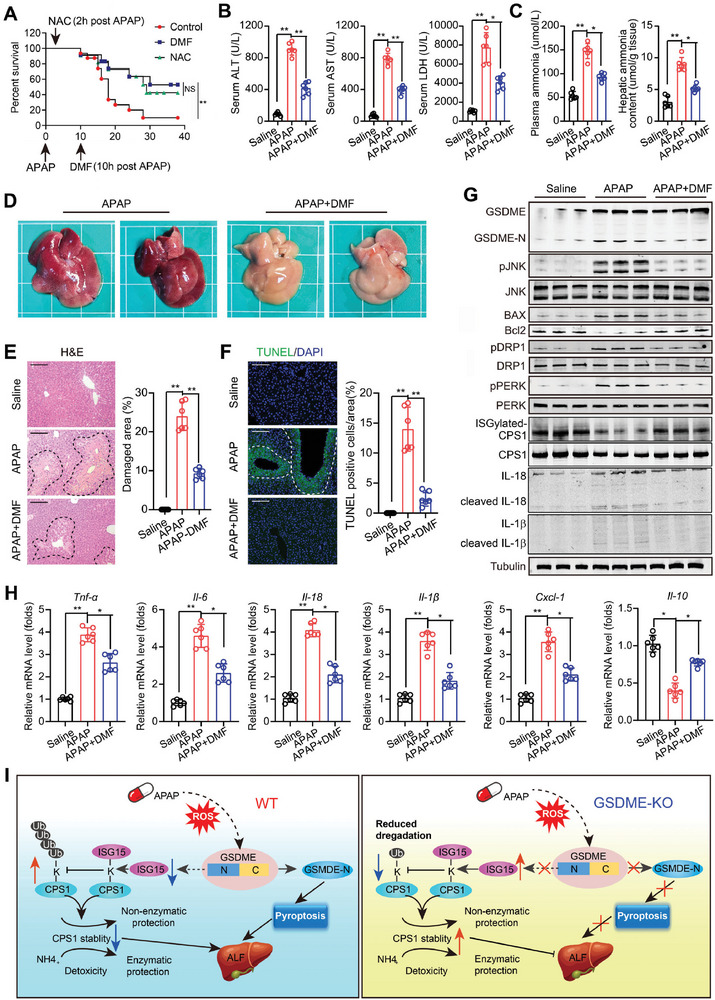
Delayed pharmacological inhibition of GSDME alleviates APAP‐related DILI. A) Survival curve of mice treated with APAP and APAP plus delayed treatment of dimethyl fumarate (APAP+DMF). DMF (30 mg k^−1^ g) was intraperitoneally injected at 10 h post APAP. To compare the therapeutic efficiency, NAC (100 mg k^−1^ g, i.p.) was administrated at 2 h post APAP. B) Serum levels of liver enzymes in mice at 24 h post‐APAP stimuli. C) Plasma and hepatic levels of ammonia in mice. D) Morphology of liver of mice. E,F) Histological analysis and fluorescent TUNEL staining in liver of mice. Scale bar = 200 µm. G) Immunoblotting analysis of GSDME, phospho‐JNK, JNK, BAX, Bcl‐2, phospho‐DRP1, DRP1, phospho‐PERK, PERK, ISGylated‐CSP1, CPS1, cleaved IL‐1β and cleaved IL‐18 in the liver of mice. H) The mRNA levels of pro‐inflammatory factors (Tnfa, Il‐6, Il‐1β, Il‐18, and Cxcl1) and anti‐inflammatory factor (Il‐10) in the liver of mice. I) A working model for the role of GSDME‐dependent pyroptosis in APAP‐related DILI. *p<0.05, **p<0.01, One‐Way ANOVA followed by Sidak's test. N = 20 in A and = 6 in B‐G.

As all the above findings pointed out that GSDME‐dependent pyroptosis is a critical pathological process in APAP‐related DILI, we thus investigated the therapeutic value of pharmacological inhibition of GSDME. Dimethyl fumarate (DMF), a pharmacological antagonist of GSDME/GSDMD,^[^ ^30^ ^]^ was administrated in mice at 10 h post‐APAP treatment, a time point exceeding the dosing window of NAC (always < 8 h). As expected, early NAC treatment within the dosing window (2 h post APAP treatment) successfully protected against APAP‐induced hepatoxicity (**Figure** **8**A). Surprisingly, delayed treatment of DMF (at 10 h post APAP treatment) exhibited comparable protection against APAP‐induced hepatoxicity to early NAC treatment (Figure 8A). At 24 h post APAP treatment, serum levels of ALT, AST, and LDH, as well as the plasma and hepatic levels of ammonia, were reduced by delayed treatment of DMF (Figure 8B,C). Morphology, histology, and fluorescent TUNEL analyses confirmed the protection of delayed treatment of DMF (Figure 8D–F). As expected, delayed treatment of DMF comprehensively inhibited the APAP‐induced changes of molecular markers in the liver (Figure 8G). Similarly, the APAP‐induced changes in mRNA levels of pro‐inflammatory factors (*Tnfa, Il‐6, Il‐1β, Il‐18*, and *Cxcl1*) and anti‐inflammatory factor (*Il‐10*) were inhibited by delayed treatment of DMF (Figure 8H). We also examined whether delayed NAC treatment (10 h post APAP) had protection in the context of GSDME blockade. Our results showed that delayed NAC treatment failed to further inhibit liver death and lower serum ALT and AST levels in APAP‐treated mice (Figure S13A–C, Supporting Information). These results indicated that pharmacological inhibition of GSDME alleviates APAP‐related DILI even beyond the conventional‐considered therapeutic window of NAC.

## 3 Discussion

Our study is the first to demonstrate the pathophysiological role of GSDME in DILI. First, we found that *Gsdme* is the only gasdermin family member induced at both transcriptional and translational levels in response to APAP stimuli. The increased cleavage of GSDME was also clearly observed in liver tissues of patients and animals with DILI. Second, we showed that deleting *Gsdme* gene protected mice from APAP‐related DILI, resulting in improved mitochondrial damage and ER stress. Using a tissue‐specific rescue mouse model, we demonstrated that GSDME in hepatocytes, rather than myeloid cells, orchestrated APAP‐induced hepatoxicity and subsequent myeloid cell infiltration and activation. Mechanistically, we identified that GSDME plays a role in APAP‐induced deISGylation and K48‐linked ubiquitination of CPS1, resulting in its downregulation and subsequent damaged ammonia metabolism. Finally, a delayed pharmacological inhibition of GSDME via administration of DMF at 10 h post‐APAP stimuli successfully alleviated APAP‐related DILI. These findings reveal a novel and unique link between GSDME‐dependent pyroptosis and ammonia metabolism during DILI (Figure 8I).

An understanding of cell death processes is crucial for effective interventions in DILI and other liver diseases. Pyroptosis, a new type of programmed cell death related to the inflammasome, has been proposed as a potential target for intervention in DILI, even before the discovery of gasdermins.^[^ ^17^ ^]^ Recent research has shown that gasdermin family proteins are the real executors of pyroptosis; however, the differences in functions among the currently known gasdermins (GSDMA, GSDMB, GSDMC, GSDMD, and GSDME)^[^ ^10^, ^11^, ^13^, ^14^, ^15^, ^16^ ^]^ are not well understood. It should be emphasized that gasdermins‐mediated pyroptosis is not necessarily harmful to health, as pyroptosis also contributes to normal physiology.^[^ ^31^ ^]^ GSDMD‐dependent canonical pyroptosis has been recently shown to protect against noninfectious liver injury by regulating apoptosis and necroptosis.^[^ ^19^ ^]^ Deletion of GSDMD can even trigger more severe sepsis‐related liver damage.^[^ ^32^ ^]^ Thus, the role of inflammasome‐related pyroptosis in liver injury has become controversial recently. Our findings suggest that only the transcription of *Gsdme* was induced post‐APAP treatment, suggesting there may be specific and varied roles of each gasdermin family member in liver disease. Our results demonstrated that global knockout of GSDME alleviates APAP‐related DILI. These findings align with observations showing that GSDME, but not GSDMD, inhibits the release of IL‐1α by TH17 cells^[^ ^33^ ^]^ and promotes apoptotic neutrophil cell lysis.^[^ ^34^ ^]^ While we observed the induction of GSDME transcription, translation, and cleavage in DILI, our findings do not definitively address whether GSDME‐mediated pyroptosis acts as the instigator of DILI. Based on our results, it is reasonable to assert that GSDME‐mediated pyroptosis could potentially contribute to the ongoing tissue damage and inflammation, possibly serving as an amplification mechanism during DILI.

Liver damage in DILI is the result of overwhelming cell death and an activated innate and adaptive immune system, compounded by other host factors such as genetics. However, the initiating site of “cell death” is not fully understood. Our findings demonstrate that rescuing GSDME expression in hepatic parenchymal cells (hepatocytes), but not in myeloid cells, in the *Gsdme*‐deficient mice, reproduced the APAP‐induced liver damage, suggesting that hepatocyte‐dependent pyroptosis signaling dominates the processes of hepatic cell death, immune cell infiltration/activation, and liver dysfunction. As hepatocytes do not express high levels of pro‐inflammatory factors, the released cell contents from lysed hepatocytes may act as triggers for immune cells to fuel hepatic inflammation. Notably. Deletion of hepatic Gsdme partially blocked the APAP‐induced depletion of inflammation‐resolving MΦ/Mo, including MerTK^+^ MΦ/Mo, TIM4^+^ KCs (ResKCs), and restorative Ly6C^lo^ IMs. These findings suggest cell‐to‐cell communication during DILI development.

Currently, it is widely accepted that the excess formation of the reactive metabolite NAPQI after an overdose of APAP leads to hepatic glutathione depletion, mitochondrial protein adducts formation and an initial oxidant stress.^[^ ^35^ ^]^ However, these events could form a vicious circle to further cause mitochondrial damage. Gasdermins including GSDME were reported to be able to pore permeabilize mitochondria to augment caspase‐3 activation during apoptosis and inflammasome activation.^[^ ^36^ ^]^ The involvement of mitochondrial damage in APAP‐induced hepatoxicity, especially in the context of pyroptosis, is rather interesting and needs further investigation. Mitochondrial function is linked to ammonia detoxification in hepatocytes.^[^ ^37^ ^]^ Extensive death of hepatocytes causes a dramatic decrease in ammonia clearance, leading to hyperammonemia and hepatic encephalopathy. Thus, dysregulated ammonia metabolism is thought to be the natural consequence of hepatocyte death in DILI. However, our results suggest that there might be more molecular mechanisms behind the accumulated ammonia in DILI. We observed a significant reduction in the global ISGylation status in the liver upon exposure to APAP stimuli. Unbiased RNA‐sequencing analysis revealed an upregulation in the mRNA level of Isg15 due to APAP stimuli, which was subsequently rescued by GSDME knockout. ISG15, a 15‐kDa ubiquitin‐like modifier involved in ISGylation, is part of a family of interferon (IFN)–stimulated effector proteins crucial during infections with various viruses. ISG15 is known to be transcriptionally regulated by factors such as IRF1,^[^ ^38^ ^]^ IRF3,^[^ ^39^ ^]^ STAT1,^[^ ^40^ ^]^ BAG3,^[^ ^41^ ^]^ ESRP1,^[^ ^42^ ^]^ etc. The regulatory influence of GSDME on the mRNA level of Isg15 remained unclear initially, given that GSDME is not a transcription factor. However, considering previous reports of GSDME interacting with IRFs or STATs,^[^ ^43^, ^44^ ^]^ and the association of another gasdermin, GSDMD, with IRFs,^[^ ^45^ ^]^ it suggests a complex molecular mechanism through which GSDME regulates *Isg15* mRNA.

Furthermore, CPS1 was identified as the most pronounced deISGylated protein in response to APAP. We also confirmed that deISGylation promoted its K48‐linked ubiquitination and subsequent degradation. As CPS1 is a liver‐specific, intramitochondrial, rate‐limiting enzyme for clearing intracellular ammonia and makes up 15%–20% of total hepatic mitochondrial protein,^[^ ^26^ ^]^ the ammonia metabolism is impaired and thus contributes to the liver injury. These results provide the first evidence that ISGylation, an uncommon type of PTM, may play a key role in the regulation of CPS1‐dependent ammonia clearance and liver damage. Notably, PTM modulation of CPS1, including acetylation, glutarylation, and O‐GlcNAcylation, etc., is an important regulatory its enzymatic activity.^[^ ^46^, ^47^, ^48^ ^]^ Interestingly, the ISGylation of CPS1 appeared to depend on GSDME. However, we lack an understanding of how GSDME influences the ISG15 and ISGylation of CPS1. Additionally, it should be noted that CPS1 was secreted from the liver to bile or blood, and exerted its protection against DILI via non‐enzymatic mechanisms.^[^ ^27^ ^]^ Thus, whether the ISGylated‐CPS1 is more stable in extracellular is an interesting issue needing further investigation.

NAC is the only approved agent that acts by generating GSH to combat the extensive ROS production with a short administration time window (< ≈8 h).^[^ ^1^ ^]^ However, the time window of NAC usage is strictly restricted because prolonged treatment with NAC is even harmful by delaying liver recovery from acetaminophen hepatotoxicity.^[^ ^49^, ^50^ ^]^ Our experimental results showed that NAC (2 h post APAP injection) successfully protected against APAP liver injury, while delayed administration of NAC (10 h post APAP injection) failed to confer protection against APAP‐hepatotoxicity in the presence of GSDME inhibitor DMF. DMF was previously found to be a potential activator of Nrf2^[^ ^51^ ^]^ but was recently discovered as an antagonist of GSDME/GSDMD.^[^ ^30^ ^]^ We found DMF treatment lowered the contents of ammonia in the liver and blood, suggesting a causal relationship between pyroptosis and ammonia metabolism. Moreover, the encouraging efficiency of DMF in delayed treatment demonstrates two things. First, the pyroptosis process may be secondary to the boosted ROS in DILI. Thus, the highly active ROS may be the ideal target for therapy of DILI in the acute phase (< 8 h) but not in the subacute phase (>8 h), as cell death are major event during this time window. Second, in addition to antioxidants, inhibiting pyroptosis or promoting the ammonia‐urea cycle may be potential avenues to treat DILI. As DMF is currently used as a formulation named “Tecfidera” for the treatment of multiple sclerosis, it is convenient to translate it to DILI treatment. The exact molecular mechanism and target of DMF may need further investigation.

In summary, our findings suggest that GSDME‐dependent pyroptosis may be involved in the DILI by controlling ISGylation of CPS1 and regulation of ammonia metabolism. These results suggest that inhibiting GSDME may be a promising therapeutic option for DILI.

## 4 Experimental Section

### Animals

Wild‐type control mice (C57BL/6J), aged 8 weeks, were procured from Sino‐British SIPPR/BK Lab Animal Ltd. (Shanghai, China). *Lysm‐Cre* mice (B6.129P2‐Lyz2tm1(cre)Ifo/J, #0 04781) and *Alb‐Cre* mice (B6.FVB(129)‐Tg(Alb1‐cre)1Dlr/J, #01 6832) were sourced from The Jackson Laboratory. The GSDME global knockout mouse (GSDME‐KO) was generated by the Shanghai Model Organisms Center (China, Shanghai) using the CRISPR/Cas9 technique. In brief, *Cas9* mRNA and gRNA were synthesized through in vitro transcription and microinjected into fertilized eggs of C57BL/6J mice to create F0 generation mice with the target gene protein reading frame. The F0 generation mice were then bred with C57BL/6J mice bearing the targeted allele of exon 4 deletion in the germline to produce GSDME‐KO mice. A *Gsdme^stop/stop^* mouse strain was generated by Cyagen Biosciences (Guangzhou, China) using CRISPR/Cas9 technology by inserting a transcriptional *Stop* element flanked by *loxP* recombination sites (*loxP‐Stop‐loxP*, LSL) upstream of the ATG start codon of *Gsdme* gene. The gRNA1 and gRNA2 to mouse *Gsdme* gene, the donor vector containing “part of 5′UTR‐loxP‐3*SV40‐Poly A ‐loxP‐part of E2” cassette, and *Cas9* mRNA were co‐injected into fertilized mouse eggs to generate targeted conditional knockin offspring (*Gsdme* ^Stop/Stop^). The *Stop* element before ATG start codon was expected to terminate the transcription of *Gsdme* gene. *Gsdme^stop/stop^* mice were crossed with *Alb‐Cre* mice or *Lysm‐Cre* mice to produce *Gsdme^stop/stop^; Alb‐Cre* mice (hepatocyte rescue of GSDME, *Gsdme^s/s^*‐HR) and *Gsdme^stop/stop^; Lysm‐Cre* mice (myeloid cell rescue of GSDME, *Gsdme^s/s^*‐MR). GSDMD global knockout mouse (GSDMD‐KO, #S‐KO‐12963) was purchased from Cyagen Biosciences.

All experimental procedures involving mice were reviewed and approved by the Institutional Animal Care and Use Committee of Shanghai Tenth People's Hospital of Tongji University (approval number: SHDSYY‐2022‐3019) and followed the Principles of Laboratory Animal Care published by the National Institutes of Health (NIH publication 86‐23 revised 1985) and ARRIVE guidelines. The mice were housed in a temperature‐controlled environment (23±2 °C) with free access to water and chow, under a 12/12‐h light/dark cycle, and every effort was made to minimize their use and discomfort. The animal health status, including body weight measurement, behavior observation, and responses to external stimuli, was monitored daily.

### Human Liver Samples

Six patients with acetaminophen (APAP)‐induced acute liver failure (ALF) were enrolled between 2020–2022 at Eastern Hepatobiliary Surgery Hospital affiliated with Naval Medical University/Second Military Medical University, Shanghai, China. These patients were diagnosed with APAP‐related DILI according to percutaneous liver biopsy. The liver tissue and blood were obtained. Patients’ clinicopathologic characteristics were analyzed, summarized, and presented in Table S1 (Supporting Information). The normal liver tissues were collected and described in the recent work.^[^ ^52^, ^53^ ^]^ The Research Ethics Committee of the Shanghai Eastern Hepatobiliary Surgery Hospital (approval number: EHBHKY2020‐K‐045) approved the research protocol. The study adhered to the ethical principles outlined in the Helsinki Declaration, and all participants provided informed consent.

### Mouse DILI Models

The study was conducted on male mice aged between 8 to 10 weeks old. Before the experiment, the mice were fasted overnight. They were then given an intraperitoneal injection of APAP (Sigma–Aldrich, UK) or saline, and were monitored at various time intervals. A recoverable APP‐DILI model was achieved by APAP injection at the dose of 350 mg k^−1^ g (i.p.). A fatal APP‐DILI model was achieved by APAP injection at the dose of 500 mg k^−1^ g (i.p.).

### Drug Administration

For DMF administration, mice were fasted overnight and then given an intraperitoneal injection of APAP. At 10 h post‐APAP treatment, the mice were given one dose of DMF (30 mg k^−1^ g, i.p.) dissolved in a mixture of 10% DMSO, 40% PEG300, 5% Tween‐80, and 45% saline. Serum and liver samples were collected at 24 h after APAP administration under sodium pentobarbital anesthesia (50 mg k^−1^ g, i.p.). For NAC treatment, NAC (100 mg k^−1^ g, i.p.) was given at 2 h (early treatment) or 10 h (delayed treatment) post‐APAP intake. For administration of L‐ornithine phenylacetate (OP), OP was administrated (0.5 g kg^−1^) at 2 h post APAP intake. Neutralizing antibodies against IL‐1β and IL‐18 (2 µg g^−1^, i.p.) were injected at 2 h post APAP to block IL‐1β and IL‐18.

### Cell Culture

The Alpha mouse liver 12 (AML12) cell line was obtained from the American Type Culture Collection (ATCC, CRL‐2254). AML12 cells were cultured in Dulbecco's Modified Eagle Medium (DMEM, Gibco) supplemented with 100 U mL^−1^ penicillin‐streptomycin and 10% fetal bovine serum (FBS, Gibco) at 37 °C in a humidified atmosphere containing 5% CO_2_. When the cells reached 80% confluence, they were detached using Trypsin‐EDTA Solution (0.25% Trypsin, 0.02% EDTA) and passed for further experiments.

### Cell Treatment

AML12 cells were incubated with APAP (20 mM) for 24 h to establish an in vitro DILI model. APAP was dissolved into serum‐free DMEM directly. Cells were seeded in six‐well plates before being subjected to treatments.

### Tissue and Blood Sampling

The mice were anesthetized with pentobarbital sodium (50 mg k^−1^ g, i.p.) and euthanized. Tissue and blood samples were collected from the mice. Blood was collected from the right atrium and allowed to clot in an upright position for at least 30 min. It was then centrifuged at 1000 × g for 10 min to obtain serum. The livers were isolated and washed with ice‐cold PBS to remove blood. The liver was then cut into pieces and rapidly frozen in liquid nitrogen for further examination.

### Biochemical Assays

Serum levels of ALT, AST, LDH, AKP, and TBA were measured using commercial assay kits obtained from Sigma–Aldrich following the manufacturer's instructions. The kits used were listed in Table S2 (Supporting Information).

### Histological Analysis

For hematoxylin‐eosin (H & E) staining, the liver tissues were embedded in paraffin and cut into sections (8 µm). The paraffin was removed with xylen and the tissues were washed with ethanol. The sections were then stained with H & E (Sigma–Aldrich) using standard procedures. The stained slides were examined under a light microscope (Leica DM2500).

### Immunohistochemistry

Immunohistochemistry was performed using commercial avidin‐biotin complex kits (Vectastain Elite ABC Kit; Vector Laboratories) as described in the previous studies.^[^ ^54^, ^55^ ^]^ The liver tissue sections, fixed in 4% paraformaldehyde, were dried at 37 °C overnight and underwent antigen retrieval. To quench endogenous peroxidase activity, 1% H_2_O_2_ was applied for 20 min, and unspecific binding was blocked with 5% normal goat serum (#31 872, Thermo Fisher) for 2 h. The sections were incubated with primary antibodies at room temperature for 4 h, followed by three washes with PBS. Biotinylated‐conjugated secondary antibodies were then applied for 1 h, and antigen‐specific immunohistochemistry was implemented using streptavidin conjugated with HRP. Chromogenic substrate diaminobenzidine was used to visualize the slides for 2 min, followed by counterstaining with hematoxylin for another 2 min. Controls were set up by omitting or preabsorbing primary antibodies and omitting secondary antibodies. The stained tissue sections were observed using an Olympus BX51 microscope (Olympus, Tokyo, Japan). The intensity of the positive area in immunohistochemistry was evaluated using Image J software. The antibodies used were listed in Table S2 (Supporting Information).

### Immunofluorescence

Immunofluorescence was performed as described in the previous studies.^[^ ^54^, ^55^ ^]^ Fixed tissues or cells were fixed in 4% paraformaldehyde (PFA) at 4 °C overnight and dehydrated in 30% sucrose. After being embedded in OCT Compound, sections (10 µm) were washed, permeabilized, and saturated with PBS, 3% BSA, 0.2% Triton X‐100, and 10% goat serum for 1 h at room temperature. The sections were then incubated with primary antibodies overnight at 4 °C. The secondary antibodies used were Alexa Fluor 488‐conjugated goat anti‐rabbit IgG and Alexa Fluor 568‐conjugated goat anti‐mouse or goat anti‐rat IgG. Nuclei were stained with DAPI, and the plasma membrane was indicated with DiO dye. The images were captured by a confocal microscope (FV1000, Olympus). The antibodies used were listed in Table S2 (Supporting Information).

### Immunoblotting

Immunoblotting was performed as described in the previous studies.^[^ ^54^, ^55^ ^]^ Tissue or cell samples were homogenized in RIPA buffer containing a protease inhibitor cocktail (Sigma) at 4°C. The homogenates were centrifuged at 14 000 × g for 20 min at 4°C. The supernatants were collected, boiled in SDS‐PAGE loading buffer, and separated by electrophoresis on SDS‐PAGE gels. The separated proteins were transferred onto nitrocellulose membranes (Millipore) using a transfer buffer. The membranes were then blocked in 5% non‐fat milk (w/v) in phosphate‐buffered saline with Tween 20, and probed with primary antibodies overnight at 4 °C, followed by secondary antibodies for 2 h at room temperature. The images were captured, and the quantitative analysis was performed using the Odyssey imaging system (Li‐Cor). The list of antibodies used was provided in Table S2 (Supporting Information).

### Immunoprecipitation

Immunoprecipitation was performed as described in the previous studies.^[^ ^56^, ^57^ ^]^ Tissue or cells were lysed in a RIPA buffer (Beyotime) containing Tris, NaCl, EDTA, DTT, β‐glycerophosphate, sodium pyrophosphate, glycerol, Triton X‐100, sodium orthovanadate, NaF, and phenylmethanesulfonyl fluoride. This buffer was supplemented with a protease inhibitor cocktail (Sigma) to prevent the degradation of proteins during lysis. After centrifugation, the lysate was used for immunoprecipitation of the protein of interest using anti‐Myc or anti‐Flag antibodies. Protein A/G plus‐agarose beads (Santa Cruz Biotechnology) were used to capture the immunocomplexes, which were then washed and subjected to immunoblotting.

### TUNEL Assay

A fluorescence terminal deoxynucleotidyl transferase–mediated deoxyuridine triphosphate nick‐end labeling (TUNEL) assay was performed with a commercial kit (ab66108) according to the manufacturer's instructions (Abcam). Images were obtained by fluorescence microscope (IX‐71; Olympus, Tokyo, Japan) with a digital camera (Olympus). Digital images were recorded and analyzed using Image J software (NIH).

### Determination of CPS1 Protein Degradation

AML12 hepatocytes were seeded in a 6‐well plate and differentiated into myotubes. Cells were serum starved for 6 h and then treated with 200 µg mL^−1^ cycloheximide (CHX). Cells were harvested at different time points after CHX treatment, and cell extracts were immunoblotted with an anti‐CPS1 antibody.

### RNA Quantification

For quantitative PCR analysis, total RNA was extracted from the samples using RNAiso Extract Reagent (Takara, Japan). The extracted RNA was reverse transcribed into cDNA using a two‐step SYBR PrimeScript RT‐PCR Kit (Takara, Japan). The PCR reaction was performed using specific primers and the CFX96 system (Bio‐Rad). The primers used in the PCR reaction were listed in the Table S2 (Supporting Information). The obtained data was normalized to the expression of *GAPDH*.

### Transcriptome RNA‐Sequencing

Transcriptome RNA‐sequencing was performed. Total RNA was isolated from the tissue using TRIzol Reagent following the manufacturer's instructions (Invitrogen) and treated with DNase I (TaKara) to remove genomic DNA. RNA quality was assessed using the Agilent 2100 Bioanalyzer and concentration was determined using the NanoDrop ND‐2000. Libraries were prepared by size‐selecting cDNA fragments of 300 bp on a 2% Low Range Ultra Agarose gel and amplifying them with Phusion DNA polymerase (NEB) for 15 PCR cycles. Library quantity was measured using TBS380, and paired‐end RNA‐seq sequencing was performed on an Illumina HiSeq 4000 sequencer. The GO pathway analysis was performed. The KEGG pathway analysis was performed using KOBAS (http://kobas.cbi.pku.edu.cn/home.do). Igraph R package tool (https://r.igraph.org/) was applied to analyze the possible gene‐network using upregulated and downregulated genes (KO‐APAP vs. WT‐APAP). The graphical representation of a pathway/network consists of the nodes (genes) and edges (biological relationship between nodes), with characteristic symbols for the different types of molecules. The raw RNA‐seq data was deposited in the NCBI Sequence Read Archive (SRA) database (SRP433975, https://www.ncbi.nlm.nih.gov/sra/?term=SRP433975).

### RNA Interference

RNA interference was used to silence ISG15 in AML12 cells using a siRNA pool (100 nM). The siRNAs were synthesized by Sangon Biotech (Shanghai, China). Lipofectamine LTX reagent (Thermo Fisher Scientific) was used to transfect the siRNA pool into AML12 cells following the manufacturer's instructions. The efficacy of the interference, which was identified by qPCR, was more than 75%.

### Plasmid Constructs and Overexpression in Cells

Recombinant vectors encoding Flag‐tagged ISG15 and Myc‐tagged CPS1 were generated by PCR‐based amplification from the cDNA of mouse hepatocyte cells and subcloned into pcDNA3.1 eukaryotic expression vectors (Invitrogen). The correctness of all constructs was verified by DNA sequencing. Transfection was performed as described above using Lipofectamine LTX reagent. Immunoblotting revealed 70%–80% transfection efficiency.

### Single‐Cell Isolation from Liver

Single cell was isolated from liver through liver perfusion and digestion. Briefly, the liver was perfused via the portal vein with HBSS (Hank's balanced salt solution) at a rate of 10 ml min^−1^ for 2 min until the liver turned pale. Then, the liver was perfused with HBSS containing 1 mg ml^−1^ Collagenase IV (Worthington Biochemical Corporation, New Jersey) at 3 ml min^−1^ for 10–15 min. The livers were minced, and the resulting tissue was incubated for 20 min with 0.5 mg ml^−1^ Collagenase IV and 0.2 mg ml^−1^ DNase on a shaker (100 rpm) at 37 °C for 30 min. All subsequent procedures were carried out at 4 °C. Samples were filtered over a 100 µm mesh filter, and red blood cells were lysed. The cells were again filtered over a 70 µm mesh filter, and hepatocytes were isolated by centrifugation at 50 g for 1 min. The remaining non‐parenchymal cells were centrifuged at 500 g for 10 min at 4 °C before proceeding to antibody staining.

### Multiplexed Flow Cytometry

The non‐parenchymal cells were isolated from the liver‐derived single‐cell solution as described previously.^[^ ^58^ ^]^ Then, the non‐parenchymal cells (1 × 106) were incubated with 0.025 µg of TruStain FcXTM anti‐CD16/32 antibody (BioLegend) for 10 min on ice to block Fc receptors. Then, the cells were stained with appropriate antibodies at 4 °C in the dark for 45 min. Dead cells were excluded using propidium Iodide (BD Biosciences) staining. The leukocyte (CD45+) clustered using multiplexing flow cytometry with antibodies against CD11b, F4/80, Ly6C, CD86, CX3CR1, CCR2, CD163 and TIM4. The KCs (CD11bintF4/80hi) clustered as ResKCs (CD11bintF4/80hiTim4+) and MoKCs (CD11bintF4/80hiTim4−). The ResKCs further clustered as CD163−‐ResKCs (CD11bintF4/80hiTim4+CD163−) and CD163+‐ResKCs (CD11bintF4/80hiTim4+CD163+). The infiltrating monocytes (IMs, CD11b^hi^F4/80^int^)‐related subsets clustered as Ly6C^+^ monocytes (CD11b^hi^F4/80^int^Ly6C^+^CD), intermediate (Int.) monocytes (CD11b^hi^F4/80^int^Ly6C^int^), Ly6C^low^monocytes (CD11b^hi^F4/80^int^Ly6C^low^). Samples were acquired using a CytoFLEX S (Beckman) and analyzed with FlowJo software (version 10; Tree Star). The list of antibodies for flow cytometry are listed in Table S2 (Supporting Information).

### Identification of ISGylated Proteins with Nano‐HPLC‐MS/MS

WT and GSDME‐KO mice were sacrificed 12 h after injection of APAP. Liver tissues were lysed and immunoprecipitated with an antibody against ISG15. The immunoprecipitated samples were separated by SDS‐PAGE to distinguish the differential band between WT‐APAP and GSDME‐KO‐APAP. The gel containing the band of interest was excised and subjected to in‐gel digestion with trypsin. The resulting peptides were analyzed by Nano‐HPnano‐LC‐MS/MS on an EASY‐nanoLC 1200 system coupled to a Q Exactive Plus mass spectrometer (Thermo Fisher Scientific, MA, USA). Tandem mass spectra were processed using PEAKS Studio version X+ (Bioinformatics Solutions Inc., Waterloo, Canada). PEAKS DB was set up to search the UniProt_Mus musculus database (version 201 907, with 22 290 entries) assuming trypsin as the digestion enzyme. Peptides with a −10lgP score greater than or equal to 20 and proteins with a −10lgP score greater than or equal to 20, and containing at least one unique peptide, were filtered for further analysis.

### Public snRNA‐seq and scRNA‐seq Data Analysis

The dataset of GSE223558 (liver tissue nuclei of healthy mice and mice with APAP [12 h post‐APAP]) and GSE188541 (human liver organoids treated with APAP) were downloaded from the GEO database. For snRNA‐seq analysis, the isolation of mouse liver nuclei was performed on n = 3 mice per time point, and the nuclei were pooled for snRNA‐seq. The analysis and processing of the single‐nuclei RNA‐seq data were performed using R 4.2.3 (R Core Team, Vienna, Austria) and RStudio software. First, doublet removal was performed by identifying clusters of cells expressing gene expression patterns of two cell types simultaneously. Then, mitochondrial quality control was conducted by removing cells with fewer than 100 detected transcripts and more than 15% mitochondrial reads. Finally, the data was log normalized and TPM‐like (base 10 000) normalized, and the analysis was carried out using the Seurat package. After applying these filtering steps, a total of 18 426 high‐quality nuclei were retained for further analysis. A principal component analysis (PCA) was performed, and the top 20 principal components (PCs) were selected for uniform manifold approximation and projection (UMAP). Shared nearest neighbors (SNN) clustering was then applied, leading to the identification of 23 distinct clusters in the data. Differential expression analysis across the clusters was conducted using the Wilcox test in the FindAllMarkers function of the Seurat R package. Marker genes for known liver cell populations were used to quantify expression levels. As a result, 10 high‐quality cell types were identified, including a cluster of Hepatocytes, a cluster of Fibroblasts, a cluster of Endothelial cells, a cluster of Hepatic stellate cells, a cluster of Kupffer cells, a cluster of Neutrophils, a cluster of Monocytes, a cluster of Macrophages, a cluster of T cells, and a cluster of B cells. Similar operations were conducted in scRNA‐seq based on GSE188541.

### Statistical Analysis

All results were presented as mean ± SEM. The normality of data distribution in all individual groups was checked using the Shapiro‐Wilk test. The sample sizes were determined according to standard protocols in the field and are indicated in the figure legend. The statistical analysis was carried out using Student's t‐test for two groups or analysis of variance (ANOVA) followed by Sidak's post‐hoc test for multiple groups. A significance level of 0.05 was considered statistically significant unless otherwise stated. GraphPad Prism 8 (GraphPad Software, San Diego, CA) was used for all statistical analyses.

## Conflict of Interest

The authors declare no conflict of interest.

## Author Contributions

S.‐X.O., J.‐H.Z., and Q.C. contributed to this work equally. P.W. and D.J.L. conceived and designed research; S.X.O., Q.C., Z.Z., Y.Z., J.W.W., S.J.S, J.H.Z., J.T.F and Y.T.C performed experiments; J.L. provided patents samples; J.T., Y.L. and F.M.S. analyzed data. S.X.O., Q.C., P.W., and D.J.L wrote the manuscript. P.W., D.J.L and J.B.Z. provided funding. All authors contributed with productive discussions and knowledge to the final version of this manuscript.

## Supporting information

Supporting Information

## Data Availability

The data that support the findings of this study are available from the corresponding author upon reasonable request.
