# Supplementary material for: Gasdermin‐E‐Dependent Non‐Canonical Pyroptosis Promotes Drug‐Induced Liver Failure by Promoting CPS1 deISGylation and Degradation
Source: Adv Sci (Weinh). 2024 Feb 28;11(16):2305715. doi: 10.1002/advs.202305715 (PMC11040357; doi:10.1002/advs.202305715)
Supplement: Supplementary file 1 — Supporting Information [file ADVS-11-2305715-s001.pdf]

ADVANCED SCIENCE
Open Access

# Supporting Information

for *Adv. Sci.*, DOI 10.1002/advs.202305715

Gasdermin-E-Dependent Non-Canonical Pyroptosis Promotes Drug-Induced Liver Failure by Promoting CPS1 deISGylation and Degradation

*Shen-Xi Ouyang, Jia-Hui Zhu, Qi Cao, Jian Liu, Zhen Zhang, Yan Zhang, Jing-Wen Wu, Si-Jia Sun, Jiang-Tao Fu, Yi-Ting Chen, Jie Tong, Yi Liu, Jia-Bao Zhang, Fu-Ming Shen, Dong-Jie Li* and Pei Wang**

# Supporting Information

## Gasdermin-E-dependent non-canonical pyroptosis promotes drug-induced liver failure by promoting CPS1 deISGylation and degradation

**Shen-Xi Ouyang[1*]**, **Jia-Hui Zhu[1*]**, **Qi Cao[2,3,4*]**, Jian Liu,[5] Zhen Zhang[1], Yan Zhang[1], Jing-Wen Wu[1], Si-Jia Sun[1], Jiang-Tao Fu[2,3,4], Yi-Ting Chen[2,3,4], Jie Tong[1], Yi Liu[1], Jia-Bao Zhang[2,3,4], Fu-Ming Shen[1], **Dong-Jie Li[1#], Pei Wang[2,3,4#]**

[1]*Department of Pharmacy, Shanghai Tenth People's Hospital, School of Medicine, Tongji University, Shanghai, China*
[2]*Department of Pharmacology, School of Pharmacy, Naval Medical University/Second Military Medical University, Shanghai, China*
[3]*Shanghai Key Laboratory for Pharmaceutical Metabolite Research, Naval Medical University/Second Military Medical University, Shanghai, China*
[4]*National Demonstration Center for Experimental Pharmaceutical Education, Naval Medical University/Second Military Medical University, Shanghai, China*
[5]*Department of Hepatic Surgery, The Eastern Hepatobiliary Surgery Hospital, Naval Medical University/Second Military Medical University, Shanghai, China*

***These authors contributed to this work equally***

**[#]Correspondence Authors:**
Prof. Pei Wang
**ORCiD:** 0000-0002-1650-3353; https://orcid.org/0000-0002-1650-3353
Ph.D., M.D., Department of Pharmacology, School of Pharmacy, Naval Medical University/Second Military Medical University, Shanghai, China
Email: pwang@smmu.edu.cn

Prof. Dong-Jie Li
**ORCiD**: 0000-0002-7119-9374; https://orcid.org/0000-0002-7119-9374
Ph.D, Department of Pharmacy, Shanghai Tenth People's Hospital, Tongji University School of Medicine, Shanghai, China
Email: djli@tongji.edu.cn

**Figure S1**

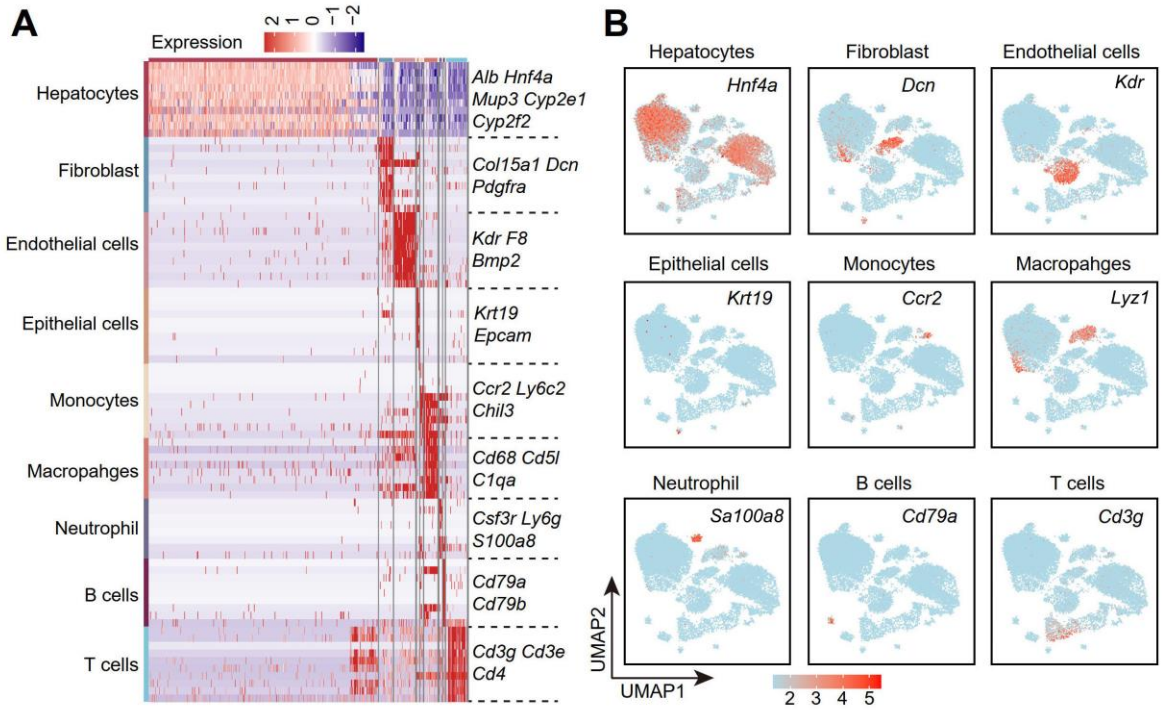


***Marker genes of snRNA-Seq analysis (GSE223558) in liver tissue of mice treated by APAP.***

**(A)** Heatmap plot showing the marker genes of 10 clusters of cells.

**(B)** Visualization of cell-type-specific marker genes of 10 clusters of cells in UMAP plot.

## Figure S2

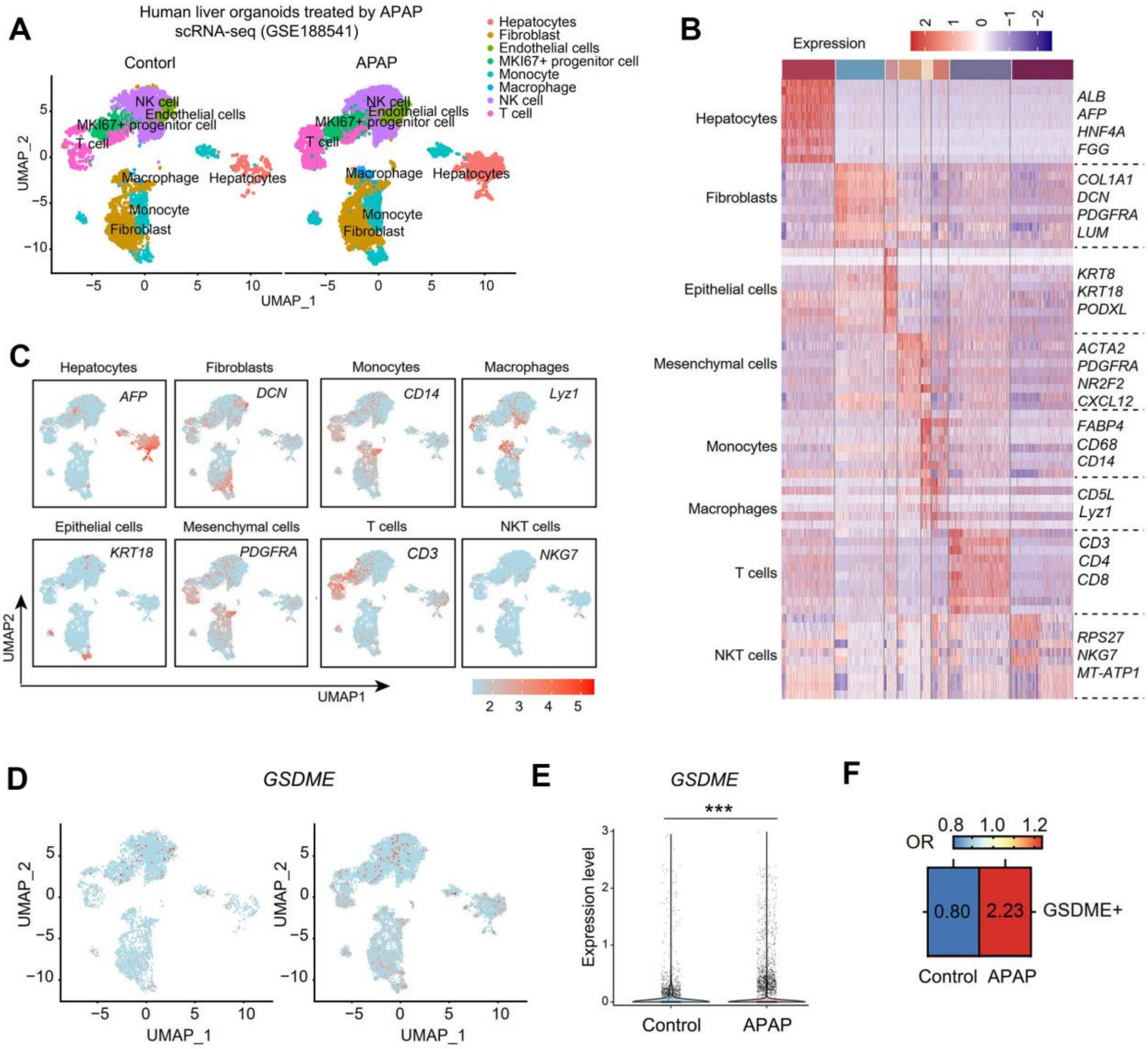


***A scRNA-Seq analysis showing the induced mRNA expression of GSDME in human liver organoids in response to APAP.***

**(A)** UMAP dimensionality reduction analysis of the scRNA-Seq data of human liver organoids treated with DMSO control or APAP (GEO accession number: GSE188541). Cells are colored by the different treatments.

(**B-C**) Heatmap and visualization of marker genes for the 8 clusters of cells.

**(D)** UMAP dimensionality reduction analysis showing the GSDME expression in cells.

**(E)** Violin plot showing the induced *GSDME* expression in response to APAP. ****P* < 0.001.

**(F)** OR plot showing the induced *GSDME* expression in response to APAP.

## Figure S3

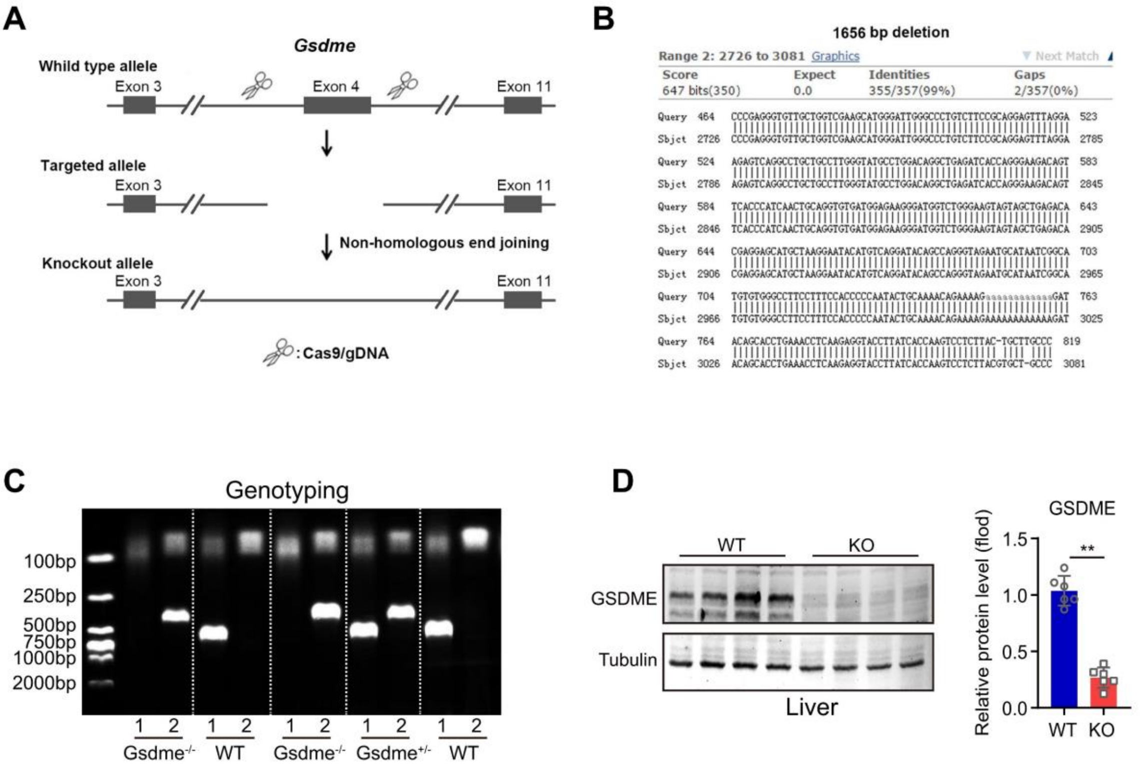


***Generation of a mouse strain with global knockout of GSDME.***

(**A**) Schematic diagram showing the knockout strategy in mice by targeting exon 4 of GSDME using CRISPR/CAS9 technology.

(**B**) Sequencing confirmation of the deletion of 1656 bp in F0 animal.

(**C**) Representative image of genotyping in F1 animals.

(**D**) Immunoblotting analysis confirmed the successful deletion of GSDME protein in GSDME-KO mice. The data were presented as means ± SEM and analyzed by two-sided unpaired Student's t-tests. $^{**}P < 0.01$. N=6 biological replicates.

## Figure S4

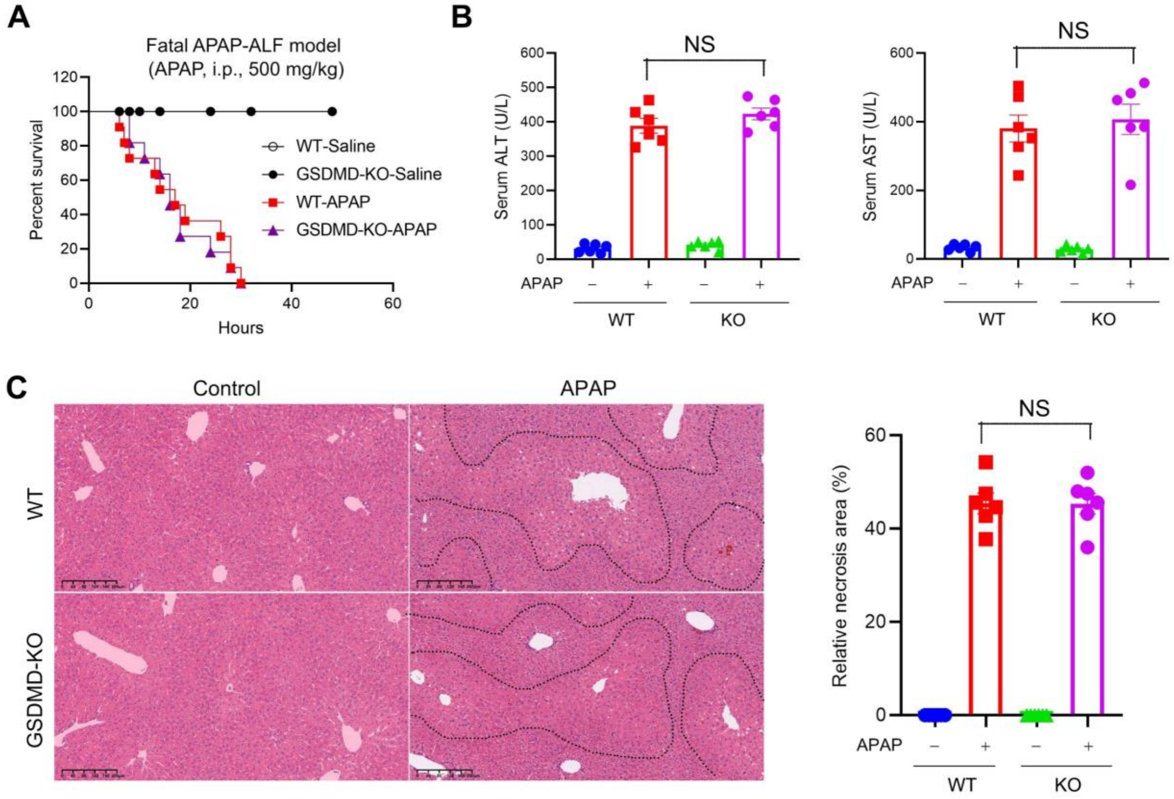


***Deletion of GSDMD in mice does not protect against APAP-induced liver injury.***

**(A)** Survival curves of WT and GSDMD-KO mice treated with a lethal dose of APAP (500 mg/kg i.p.).

**(B)** Serum ALT and AST levels in WT and GSDMD-KO mice treated with APAP in a coverable model (i.p., 350 mg/kg).

**(C)** H & E staining of liver tissue of WT and GSDMD-KO mice treated with APAP in a coverable model (i.p., 350 mg/kg). Scale bar = 200 μm.

One-way ANOVA followed by Sidak’s test. N=6 biological replicates. NS, no significance.

**Figure S5**

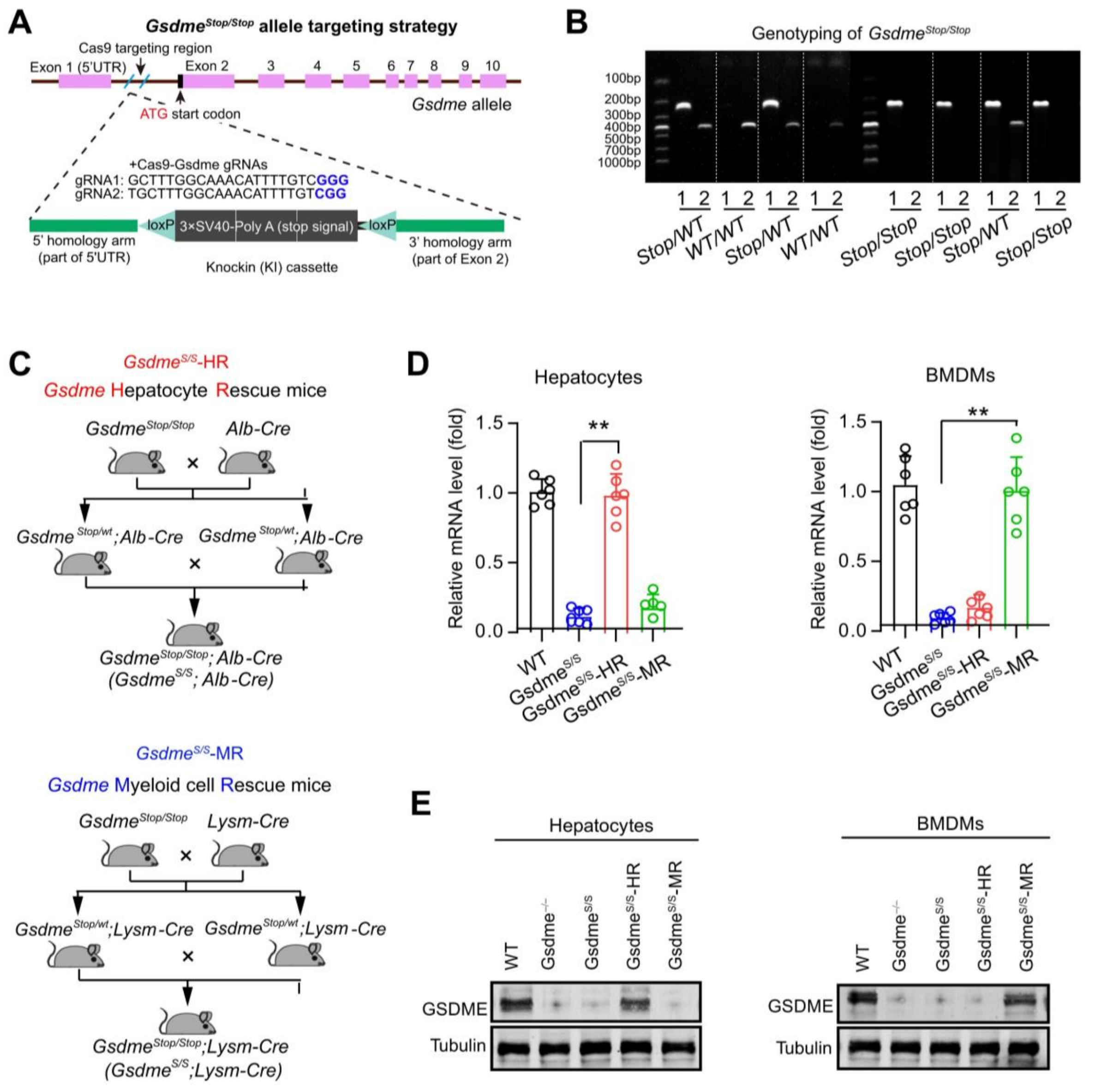


***Generation of mouse stains with conditional rescue of GSDME in cardiomyocytes and myeloid cells respectively.***

(**A**) Schematic diagram showing the gene targeting strategy for generation of a mouse strain carrying a transcriptional *Stop* element flanked by *loxP* recombination sites (*loxP*-*Stop*-*loxP*, LSL) upstream of the ATG start codon of *Gsdme* gene. The gRNA1 and gRNA2 to mouse *Gsdme* gene, the donor vector containing "part of 5'UTR-loxP-3*SV40-Poly A -loxP-part of E2" cassette, and *Cas9* mRNA were co-injected into fertilized mouse eggs to generate targeted conditional knockin offspring (*Gsdme*$^{\text{Stop/Stop}}$). The sequences of gRNA1 and gRNA2 were also shown. The *Stop* element before ATG start codon was expected to terminate the transcription of *Gsdme* gene.

(**B**) Genotyping of *Gsdme*$^{\text{Stop/Stop}}$ mice.

(**C**) The *Gsdme*$^{\text{Stop/Stop}}$ mouse strain was crossed with *Alb*-Cre or *Lysm*-Cre mouse to produce *Gsdme*$^{\text{Stop/Stop}}$;*Alb*-Cre mouse (hepatocyte rescue of GSDME, referred as *Gsdme*$^{\text{S/S}}$-HR) or *Gsdme*$^{\text{Stop/Stop}}$;*Lysm*-Cre mouse (myeloid cell rescue of GSDME, referred as *Gsdme*$^{\text{S/S}}$-MR). The Cre expression specific in hepatocyte or myeloid cells can delete the *Stop* element to

allow GSDME re-transcription.

(**D**) Quantitative PCR analysis showing *Gsdme* mRNA level in hepatocytes and bone-marrow-derived macrophages (BMDMs) from WT, *Gsdme*$^{-/-}$, *Gsdme*$^{Stop/Stop}$, *Gsdme*$^{S/S}$-HR and *Gsdme*$^{S/S}$-MR mice. $n = 6$ biologically independent experiments. The data were presented as means ± SEM and analyzed by two-sided unpaired Student's t-tests. $^{**}P < 0.01$.

(**E**) Immunoblotting analysis of GSDME protein level in hepatocytes and bone marrow from WT, *Gsdme*$^{-/-}$, *Gsdme*$^{Stop/Stop}$, *Gsdme*$^{S/S}$-HR and *Gsdme*$^{S/S}$-MR mice. GSDME protein was rescued in hepatocytes of *Gsdme*$^{S/S}$-HR mice and BMDMs of *Gsdme*$^{S/S}$-MR mice. Tubulin was used as a loading control.

**Figure S6**

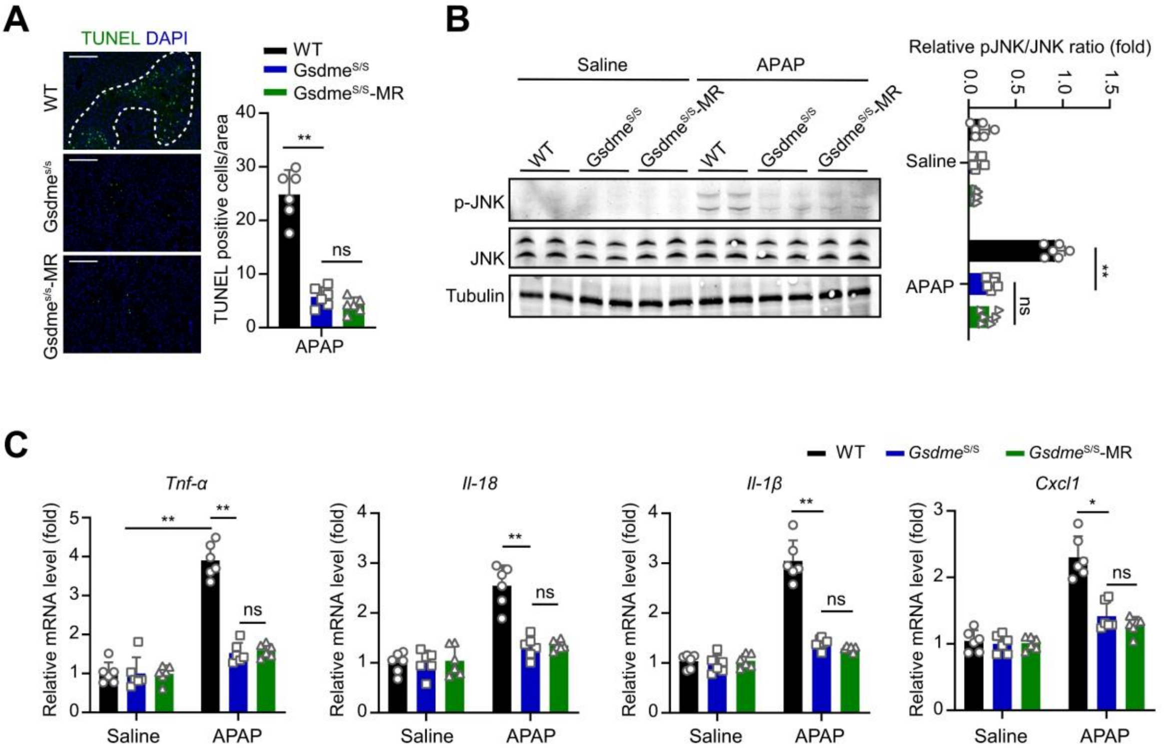


***Rescue of GSDME in myeloid cells failed to reproduce APAP-induced liver damage.***

(**A**) Representative TUNEL immunofluorescence staining and quantification analyses were performed in liver tissue of WT, *Gsdme*$^{s/s}$ and *Gsdme*$^{s/s}$-MR mice treated with APAP. Dotted line content indicates the area of TUNEL-positive cells. N = 6 biological replicates per group. Scale bar = 200 μm.

(**B**) Immunoblotting and quantification analyses of phospho-JNK (p-JNK) and JNK in liver tissues of WT, *Gsdme*$^{s/s}$ and *Gsdme*$^{s/s}$-MR mice treated with saline or APAP. N = 6 biological replicates per group.

(**C**) The mRNA levels of pro-inflammatory factors (*Tnf*$\alpha$, *Il18, and Il1*$\beta$) and chemokines (*Cxcl1*) in liver tissue of WT, *Gsdme*$^{s/s}$ and *Gsdme*$^{s/s}$-MR mice treated with saline or APAP. N = 6 biological replicates per group.

*P<0.05, **P<0.01, NS, no significant difference. One-way ANOVA followed by Sidak's test. N=6 biological replicates.

**Figure S7**

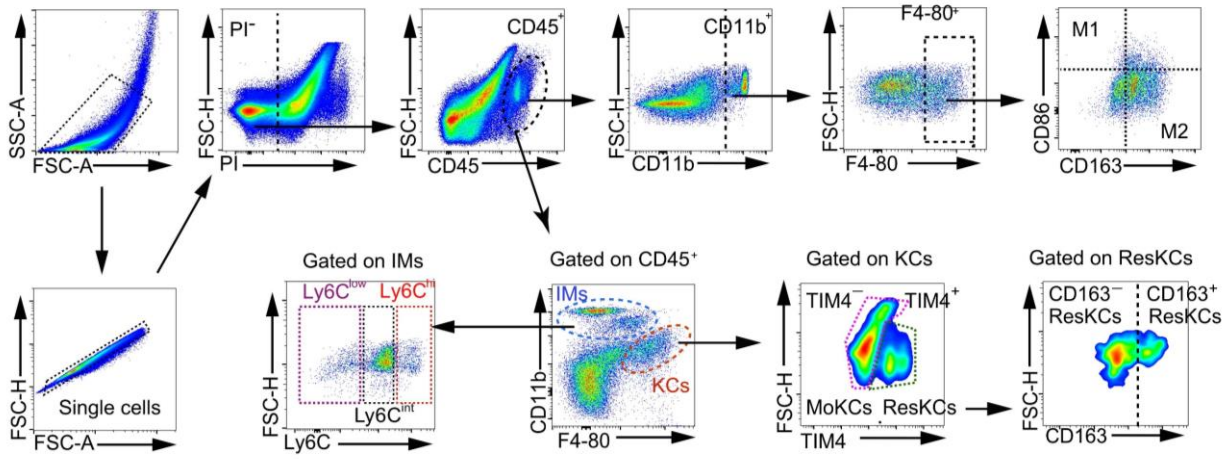


***Gating strategy for the identification of distinct myeloid cell subsets in the liver tissues of WT, Gsdme$^{S/S}$, or Gsdme$^{S/S}$*-HR *mice.***

**Figure S8**

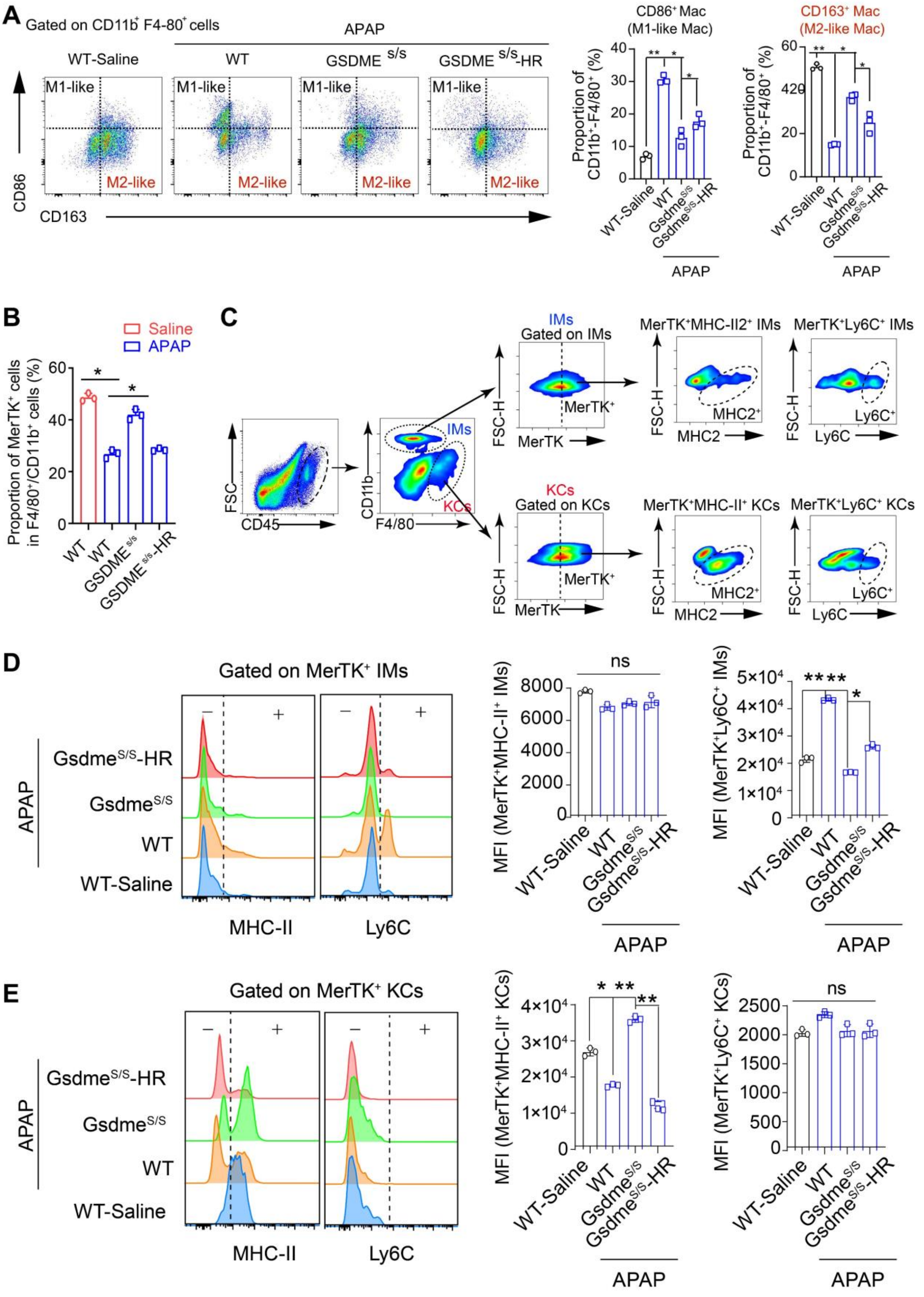

A
Gated on CD11b+ F4-80+ cells
APAP
WT-Saline
WT
GSDME s/s
GSDME s/s-HR
M1-like
M2-like
CD86
CD163
CD86+ Mac (M1-like Mac)
CD163+ Mac (M2-like Mac)
Proportion of CD11b+-F4/80+ (%)
WT-Saline
WT
Gsdme S/S
Gsdme S/S-HR
APAP
B
Saline
APAP
Proportion of MerTK+ cells in F4/80+/CD11b+ cells (%)
WT
WT
GSDME s/s
GSDME s/s-HR
C
FSC
CD45
CD11b
F4/80
IMs
KCs
Gated on IMs
Gated on KCs
FSC-H
MerTK
MerTK+
MerTK+MHC-II2+ IMs
MerTK+Ly6C+ IMs
MerTK+MHC-II+ KCs
MerTK+Ly6C+ KCs
MHC2
MHC2+
Ly6C
Ly6C+
D
Gated on MerTK+ IMs
APAP
Gsdme S/S-HR
Gsdme S/S
WT
WT-Saline
MHC-II
Ly6C
MFI (MerTK+MHC-II+ IMs)
MFI (MerTK+Ly6C+ IMs)
ns
E
Gated on MerTK+ KCs
MFI (MerTK+MHC-II+ KCs)
MFI (MerTK+Ly6C+ KCs)

***Hepatocyte GSDME regulates M1/M2 polarization and antigen-presenting function in macrophages upon APAP stimuli.***

(**A**) Representative FACS plots and quantification analyses on the proportion of M1-like ($CD86^+$) and M2-like ($CD163^+$) macrophage in macrophage ($CD11b^+F4/80^+$) in liver tissues of WT, *Gsdme*$^{s/s}$ and *Gsdme*$^{s/s}$-HR mice treated with saline or APAP.

(**B**) The proportion of $MerTk^+$ macrophages in $F4/80^+CD11b^+$ macrophages in four groups of mice upon APAP stimuli.

(**C**) Gating strategy of flow cytometry to determine the proportion of MHC-$II^+$ and $Ly6C^+$ cells within the $MerTk^+$ macrophages KCs and IMs respectively. MHC-$II^+$ macrophages have antigen-presenting and inflammatory-resolving activity, whereas Ly6C+ cells have potent pro-inflammatory activity.

(**D**) The expression level of MHC-II and Ly6C in IMs. MFI: mean fluorescence intensity.

(**E**) The expression level of MHC-II and Ly6C in KCs. MFI: mean fluorescence intensity.

**P*<0.05, ***P*<0.01, One-Way ANOVA followed by Sidak's test. N=3 biological replicates.

# Figure S9

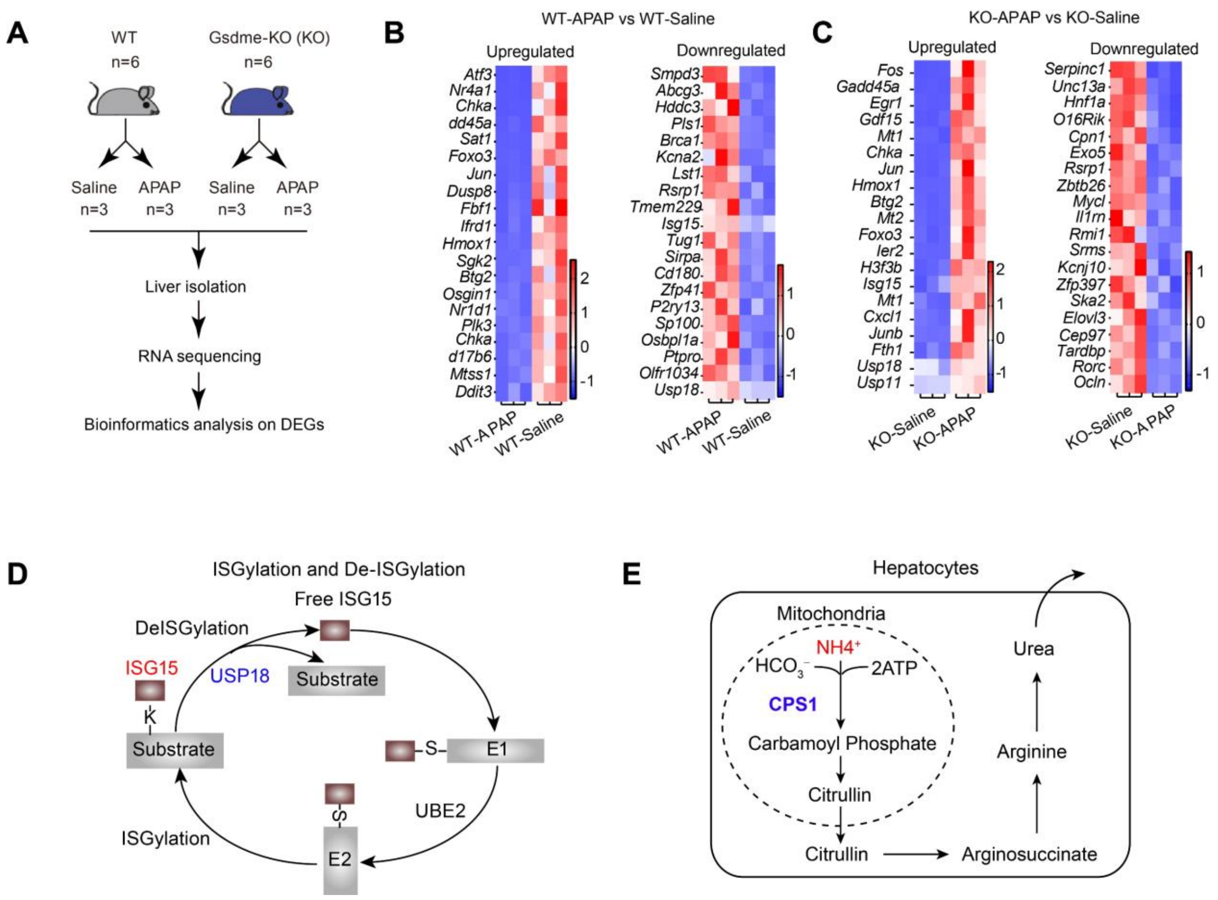


***Transcriptome RNA-sequencing identifies the alteration of ISG15-USP18 signaling in APAP-ALF.***

(**A**) Schematic of experimental design of transcriptome RNA-sequencing in mice.

(**B-C**) Heatmap derived from RNA-sequencing analysis indicating the differentially expressed genes (DEGs) between WT mice or KO mice liver tissues treated with Saline or APAP. Red, upregulated; Blue, downregulated.

(**D**) Schematic diagram showing the ISGylation and De-ISGylation.

(**E**) Schematic diagram showing the urea cycle in hepatocytes.

## Figure S10

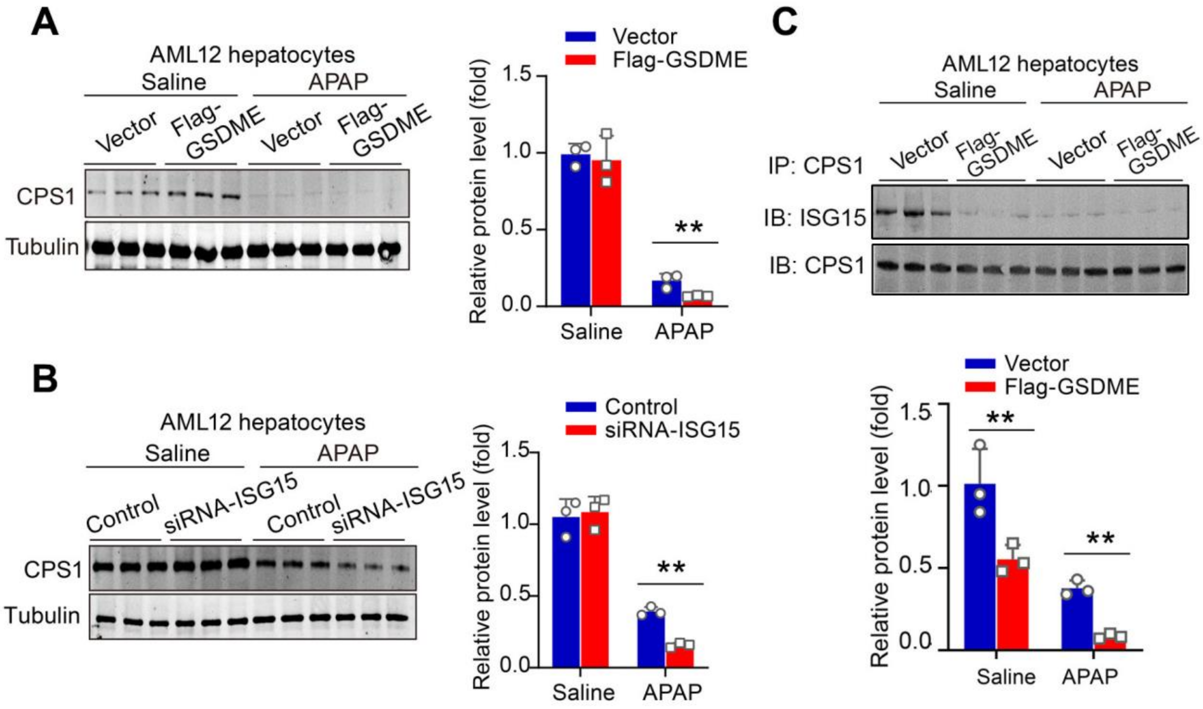


***Modulation of GSDME and ISG15 affects total CPS1 protein and ISGylated-CPS1.***

(**A**) Immunoblotting and quantification analyses of CPS1 in AML12 cells transfected with vector or FLAG-GSDME and treated with saline or APAP. N = 3 biological replicates per group.

(**B**) Immunoblotting and quantitative analysis of CPS1 in AML12 cells transfected with or without siRNA-mediated ISG15 knockdown and treated with saline or APAP. N = 3 biological replicates per group.

(**C**) Immunoblotting and quantification analyses of ISGylated-CPS1 in AML12 cells transfected with vector or Flag-tagged GSDME and treated with saline or APAP. N = 3 biological replicates per group.

**P*<0.05, ***P*<0.01, One-Way ANOVA followed by Sidak's test. N=3-6 biological replicates.

**Figure S11**

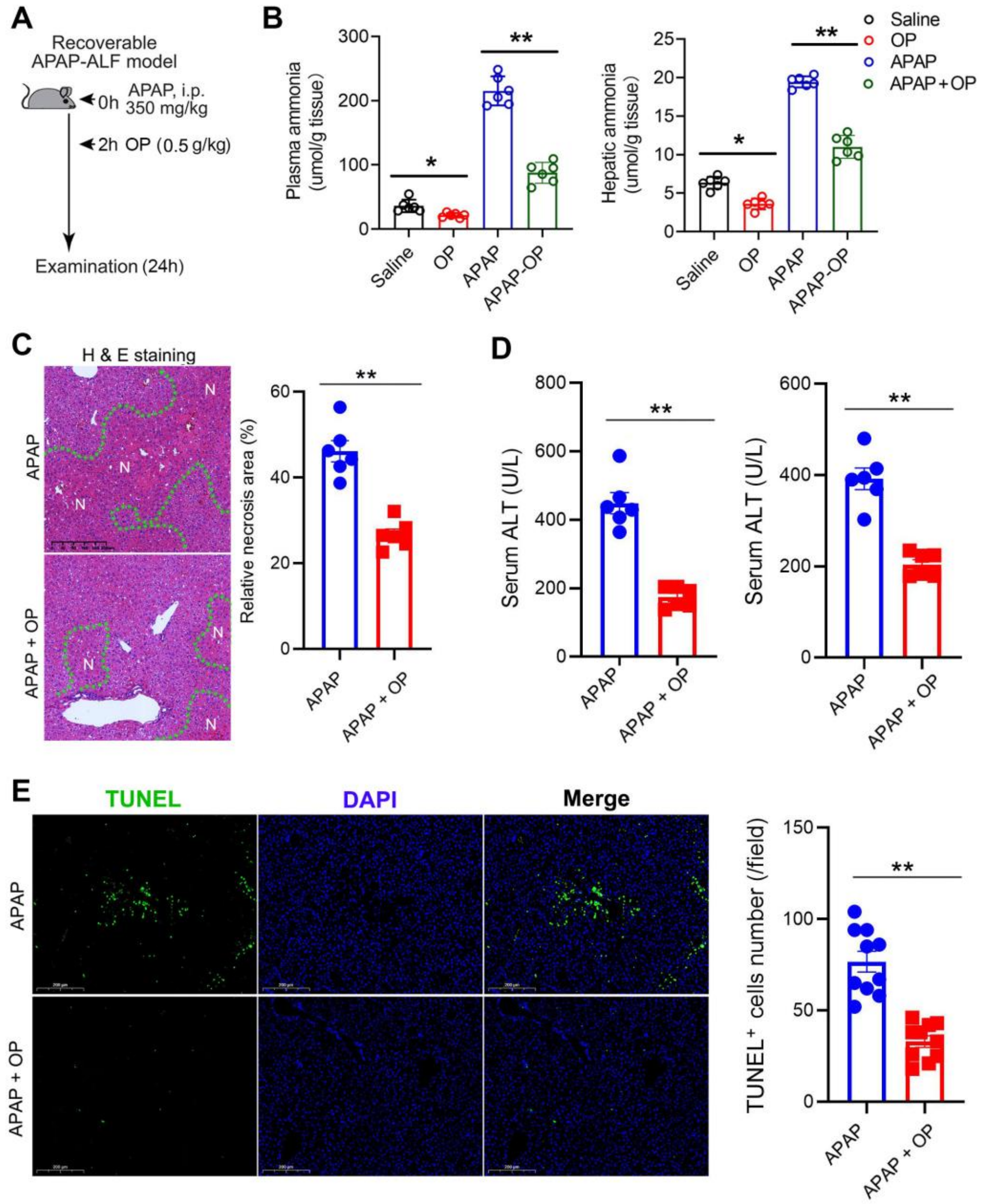


**Pharmacologically clearance of ammonia protects against APAP-induced liver injury.**

(**A**) L-ornithine phenylacetate (OP), a well-established ammonia-clearing agent, was administrated (0.5 g/kg) at 2 hours post APAP or saline injection.

(**B**) Serum and intrahepatic ammonia levels in control and APAP-injected mice receiving OP treatment.

(**C**) H & E staining of liver tissue in APAP-injected mice and APAP-injected mice receiving OP treatment. Scale bar = 200 μm.

(**D**) Serum ALT and AST levels in APAP-injected mice and APAP-injected mice receiving OP treatment.

(**E**) TUNEL staining of liver tissue in APAP-injected mice and APAP-injected mice receiving OP treatment. Scale bar = 200 μm.

**P*<0.05, ***P*<0.01, One-Way ANOVA followed by Sidak's test. N=6 biological replicates.

**Figure S12**

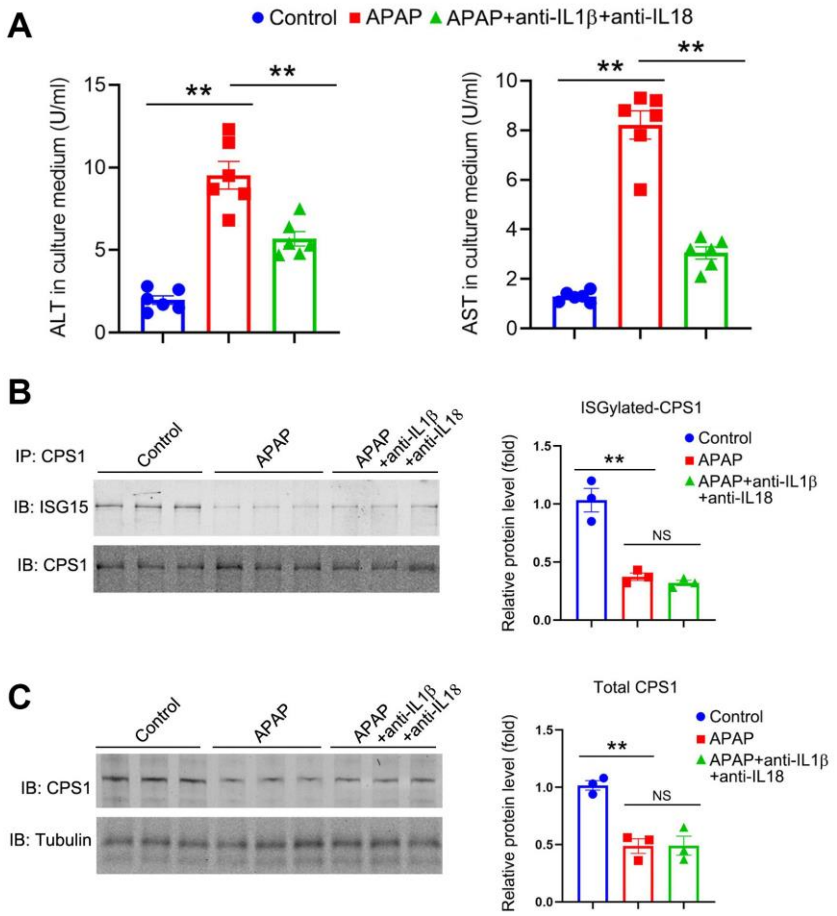


**Neutralizing antibodies against IL-1β and IL-18 protect against APAP-induced liver injury but fail to block CP1 deISGylation *in vivo*.**

(**A**) Serum ALT and AST levels in mice treated by APAP and APAP plus anti-IL-1β/anti-IL-18.

(**B**) ISGylated-CPS1 protein level in liver of mice treated by APAP and APAP plus anti-IL-1β/anti-IL-18.

(**C**) Total CPS1 protein level in liver of mice treated by APAP and APAP plus anti-IL-1β/anti-IL-18.

**P*<0.05, ***P*<0.01, One-Way ANOVA followed by Sidak's test. N=6 biological replicates.

**Figure S13**

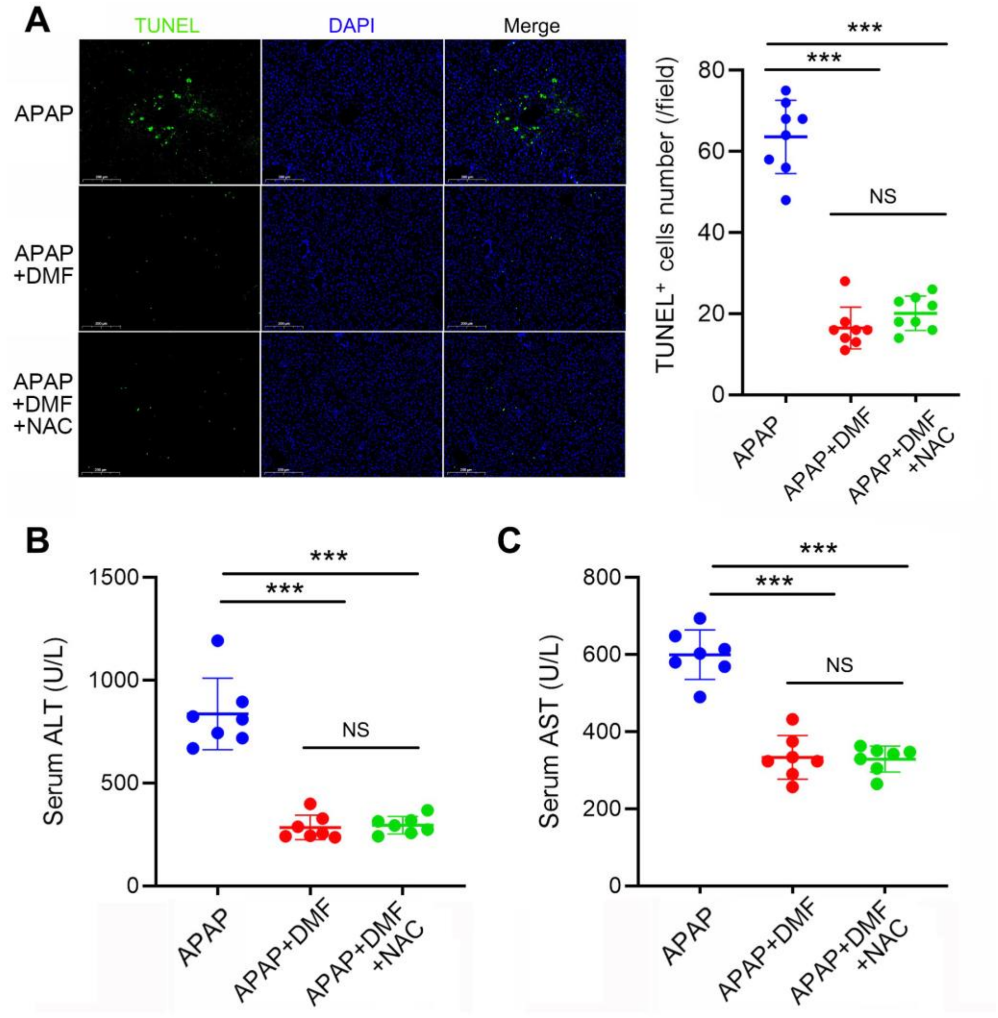


**Delayed administration of NAC at 10 hours post-APAP does not provide further protection in the presence of GSDME blockade.**

**(A)** TUNEL staining showing the effects of DMF (10 h post-APAP) or DMF+NAC (10 h post-APAP) on liver cell death. Scale bar = 200 μm.

**(B-C)** Effects of DMF (10 h post-APAP) or DMF+NAC (10 h post-APAP) on serum ALT and AST levels in mice.

**P*<0.05, ***P*<0.01, One-Way ANOVA followed by Sidak's test. N=6 biological replicates.

**Table S1: Individual information of patients with APAP-ALF utilized for the study**

| Patient | Sex | Age | Diagnosis method | Patient history | Tobacco | Alcohol | Time post overdose-APAP intake | ALT (U/L) | AST (U/L) | TBIL |
|---|---|---|---|---|---|---|---|---|---|---|
| 1 | Male | 45 | Percutaneous liver biopsy | Simple fatty liver | Y | Y | 14 | 258.4 | 351.7 | 5.8 |
| 2 | Female | 47 | Percutaneous liver biopsy | Simple fatty liver; Hypertension | N | N | 8 | 428.5 | 426.8 | 6.8 |
| 3 | Male | 57 | Percutaneous liver biopsy | Hypertension | Y | Y | 11 | 373.9 | 374.4 | 7.2 |
| 4 | Female | 52 | Percutaneous liver biopsy | Obesity | N | Y | 12 | 274.9 | 248.1 | 5.3 |
| 5 | Male | 48 | Percutaneous liver biopsy | Cholecystitis | N | Y | 6 | 318.6 | 305.9 | 5/8 |
| 6 | Male | 36 | Percutaneous liver biopsy | Hyperlipidemia | N | Y | 10 | 283.7 | 231.5 | 6.3 |

## Table S2. Key Reagents

| REAGENT | SOURCE | IDENTIFIER |
|---|---|---|
| **Antibodies** | | |
| Rabbit monoclonal anti-IL-6 | Abcam | CAT#ab233706, RRID:AB_2889391 |
| Rabbit monoclonal anti-GSDME | Abcam | CAT#ab215191, PRID: AB_2737000 |
| Rabbit monoclonal anti-Caspase8 | Abcam | CAT# ab108333, PRID: AB_10866391 |
| Rabbit monoclonal anti-Caspase9 | Abcam | CAT# ab32539, PRID: AB_725960 |
| Rabbit monoclonal anti-Drp1 | Abcam | CAT# ab184247, PRID: AB_2895215 |
| Rabbit monoclonal anti-Ubiquitin | Abcam | CAT# ab134953, PRID: AB_2801561 |
| Rabbit monoclonal anti-Ubiquitin(K48) | Abcam | CAT# ab140601, PRID: AB_2783797 |
| Rabbit monoclonal anti-Ubiquitin(K63) | Abcam | CAT# ab179434, PRID: AB_2895239 |
| Rabbit monoclonal anti-pPERK | Cell signaling technology | CAT# 3179S, PRID: AB_2095853 |
| Mouse monoclonal anti-F4/80 | Santa Cruz Biotechnology | CAT#sc-52664, RRID:AB_629466 |
| Mouse monoclonal anti-IL-1β | Santa Cruz Biotechnology | CAT#sc-52012, RRID:AB_629741 |
| Mouse monoclonal anti-EndoG | Santa Cruz Biotechnology | CAT#sc-365359, RRID: AB_10843802 |
| Mouse monoclonal anti-COX4 | Santa Cruz Biotechnology | CAT#sc-376731, RRID: AB_2904544 |
| Mouse monoclonal anti-Cyt-C | Santa Cruz Biotechnology | CAT#sc-13156, RRID: AB_627385 |
| Rabbit polyclonal anti-CPS1 | Proteintech | CAT# 18703-1-AP, PRID: AB_2084238 |
| Rabbit polyclonal anti-ISG15 | Proteintech | CAT#15981-1-AP, RRID:AB_2126302 |
| Rabbit polyclonal anti-pDrp1 | Proteintech | CAT# 12957-1-AP, PRID: AB_2093525 |
| Rabbit polyclonal anti-MFN2 | Proteintech | CAT#12186-1-AP, PRID: AB_2266320 |
| Mouse monoclonal anti-JNK | Proteintech | CAT#66210-1-Ig, PRID: AB_2881601 |
| Mouse monoclonal anti-pJNK | Proteintech | CAT#80024-1-RR, PRID: AB_2882943 |

| | | |
|---|---|---|
| Mouse monoclonal anti-IL-18 | Proteintech | CAT#60070-1-Ig, PRID:AB_2280158 |
| Mouse monoclonal anti-Caspase3 | Proteintech | CAT#66470-2-Ig, PRID: AB_2876892 |
| Rabbit polyclonal anti-BAX | Proteintech | CAT#50599-2-Ig, PRID: AB_2061561 |
| Rabbit monoclonal anti-Bcl2 | Proteintech | CAT# 68103-1-Ig, PRID: AB_2923635 |
| Rabbit polyclonal anti-AIF | Proteintech | CAT# 17984-1-AP, PRID: AB_2224539 |
| Mouse monoclonal anti-Tubulin | Proteintech | CAT#66031-1-Ig, RRID:AB_11042766 |
| Mouse monoclonal anti-Flag | Sigma-Aldrich | CAT#F1804, RRID: AB_262044 |
| Mouse monoclonal anti-Myc | Sigma-Aldrich | CAT# M4439, RRID: AB_439694 |
| Mouse monoclonal anti-F4/80-APC R700 | BD Biosciences | CAT#565787, RRID: AB_2869711 |
| Rat monoclonal anti-CD45-BV605 | BioLegend | CAT#103139, RRID: AB_2562341 |
| Rat monoclonal anti-CD11b-FITC | BioLegend | CAT#101205, RRID: AB_312788 |
| Rat monoclonal anti-TIM4-PerCP Cy5 | BioLegend | CAT#130019, RRID: AB_2876458 |
| Mouse monoclonal anti-CX3CR1-BV421 | BioLegend | CAT# 49023, RRID: AB_2565706 |
| Rat monoclonal anti-Ly6C-PE | BioLegend | CAT#128007, RRID: AB_1186133 |
| Rat monoclonal anti-CD163-PE/Cyanine7 | BioLegend | CAT#155319, RRID: AB_2890710 |
| Mouse monoclonal anti-CD86-BV510 | BioLegend | CAT#105039, RRID: AB_2562370 |
| Rat monoclonal anti-CCR2-BV650 | BioLegend | CAT#150613, RRID: AB_2721553 |
| APC anti-mouse MERTK (Mer) Antibody | BioLegend | CAT#151508, RRID: AB_2650739 |
| APC/Fire™ 750 anti-mouse I-A/I-E Antibody | BioLegend | CAT# 107652, RRID: AB_2616729 |
| Propidium Iodide Solution | BD Biosciences | CAT# 556463, RRID: AB_2869075 |
| Goat Polyclonal IRDye 800CW Goat anti-mouse IgG antibody | LI-COR Biosciences | CAT#926-32210, RRID: AB_621842 |
| Goat Polyclonal IRDye 800CW Goat anti-rabbit IgG antibody | LI-COR Biosciences | CAT#925-32211, RRID: AB_2651127 |

| | | |
|---|---|---|
| Goat Polyclonal Alexa Fluor 488-conjugated anti-rabbit IgG | Thermo Fisher Scientific | CAT#A-11034, RRID:AB_2576217 |
| Goat Polyclonal Alexa Fluor 568-conjugated anti-rat IgG | Thermo Fisher Scientific | CAT#A-11077, RRID:AB_2534121 |
| Goat Polyclonal Alexa Fluor 568-conjugated anti-mouse IgG | Thermo Fisher Scientific | CAT#A-21134, RRID:AB_2535773 |
| **Biological Samples** | | |
| Human liver tissue samples from ALI patients | Eastern Hepatobiliary Surgery Hospital, Naval Medical University, Shanghai, China | Approval number: EHBHKY2020-K-045 |
| **Chemicals, Peptides, and Recombinant Proteins** | | |
| BSA | Roche | 03117405001 |
| Palmitic acid | Sigma-Aldrich | P9767 |
| Protease inhibitor cocktail | Sigma-Aldrich | P8340 |
| Protein A/G PLUS-Agarose | Santa Cruz Biotechnology | sc-2003 |
| DEPC-treated water | Thermo Fisher Scientific | 4387937 |
| DiO dye | Thermo Fisher Scientific | V22886 |
| DAPI | Thermo Fisher Scientific | D1306 |
| Mito-ROS | AAT Bioquest | 16052 |
| TMRE | Sigma-Aldrich | MAK146 |
| Propidium Iodide Solution | BD Biosciences | 556463 |
| Normal goat serum | Thermo Fisher Scientific | 31872 |
| Formalin | Thermo Fisher Scientific | 23-305-510 |
| Lipofectamine LTX | Thermo Fisher Scientific | 15338-100 |
| DMEM/F-12 Medium | Thermo fisher scientific | 11320033 |
| 1640 Medium | Thermo fisher scientific | 11875085 |

| | | |
|---|---|---|
| Fetal bovine serum | Thermo fisher scientific | 10099141 |
| Penicillin-Streptomycin | Thermo fisher scientific | 15070063 |
| Pre-stained Protein Marker | Thermo fisher scientific | 26616 |
| RIPA buffer | Beyotime | P0013B |
| Prestained Protein Marker | GeneTex | GTX50875 |
| Acetaminophen (APAP) | MedChemExpress LLC | HY-66005 |
| Nitrocellulose membrane | Millipore | 1620112 |
| Collagenase IV | Worthington | LS004186 |
| DL1000 DNA ladder | Takara Bio | 3591A |
| DL10000 DNA ladder | Takara Bio | 3584A |
| RNAiso Extract Reagent | Takara Bio | 9109 |
| Dimethyl fumarate (DMF) | Selleck | S2586 |
| NAC | Sigma | A9165 |
| Neutralizing antibodies against IL-1β | Proteintech | 26048-1-AP |
| Neutralizing antibodies against IL-18 | Proteintech | 10663-1-AP |
| **Critical Commercial Assays** | | |
| Plasma Membrane Protein Isolation kit | Abcam | ab65400 |
| PrimeScript™ RT-PCR Kit | Takara Bio | RR014A |
| LDH assay Kit | Sigma-Aldrich | 11644793001 |
| ALT assay Kit | Sigma-Aldrich | MAK052 |
| AST assay Kit | Sigma-Aldrich | MAK055 |
| Bile acid assay KIt | Sigma-Aldrich | MAK309 |
| SOD assay Kit | Sigma-Aldrich | 19160 |
| TUNEL kit | Abcam | ab66108 |
| AKP assay KIt | Beyotime Biotechnology | P0321S |
| BCA protein assay kit | Beyotime Biotechnology | P0011 |
| Fast Silver Stain Kit | Beyotime Biotechnology | P0017S |
| MitoSOX probe | Thermo Fisher Scientific | M36008 |
| AxyPrep DNA GelExtraction Kit | Axygen Biosciences | #AP-GX-250 |

| | | |
|---|---|---|
| Blood Ammonia Content Assay Kit | Solarbio Life science | BC4385 |
| **Experimental Models: Cell Lines** | | |
| Alpha mouse liver 12 | ATCC | #CRL-2254, RRID:CVCL_0140 |
| **Deposited data** | | |
| RNA-seq on livers from WT and GSDME-KO mice injected APAP | This paper | SRA: SRP433975 |
| **Experimental Models: Organisms/Strains** | | |
| Mouse: C57BL/6J | Shanghai Sino-British SIPPR/BK Lab Animal Ltd. | N/A |
| Mouse: *Lysm*-Cre: B6.129P2-*Lyz2*$^{tm1(cre)Ifo/}$J | Jackson Laboratory | JAX stock 004781 |
| Mouse: *Alb*-Cre: B6.FVB(129)-Tg(Alb1-cre)1Dlr/J | Jackson Laboratory | JAX stock 016832 |
| Mouse: GSDME-KO | Shanghai Biomodel Organism Science & Technology Development Co., Ltd. | N/A |
| Mouse: *Gsdme*$^{stop/stop}$ | Cyagen Bioscience Inc. | KICMAS190710LY2 |
| Mouse: GSDMD | Cyagen Bioscience Inc. | S-KO-12963 |
| **Oligonucleotides** | | |
| Primers for human *GSDME* Forward | Sangon Biotech | GATGCTGATGGTGACCTGATTG |
| Primers for human *GSDME* Reverse | Sangon Biotech | GCCAAGGGTGAGGGATAAAA |
| Primers for human *CASPASE3* Forward | Sangon Biotech | TCTGACTGGAAAGCCGAAACT |
| Primers for human *CASPASE3* Reverse | Sangon Biotech | GACTGGATGAACCACGACCC |

| | | |
|---|---|---|
| Primers for human *IL18* Forward | Sangon Biotech | ACCTGGAATCAGATTACTTTGGC |
| Primers for human *IL18* Reverse | Sangon Biotech | TATCATGTCCTGGGACACTTCTC |
| Primers for human *GAPDH* Forward | Sangon Biotech | CCCATCACCATCTTCCAG |
| Primers for human *GAPDH* Reverse | Sangon Biotech | ATGGGGAAGGTGAAGGTCG |
| Primers for mouse *Cxcl1* Forward | Sangon Biotech | CACCCAAACCGAAGTCATAGC |
| Primers for mouse *Cxcl1* Reverse | Sangon Biotech | GGGGACACCTTTTAGCATCTTT |
| Primers for mouse *Ccl2* Forward | Sangon Biotech | TGAGGTGGTTGTGGAAAAGG |
| Primers for mouse *Ccl2* Reverse | Sangon Biotech | CCGTAGCGTTGGGTTTCT |
| Primers for mouse *Ccl5* Forward | Sangon Biotech | ACTCCCTGCTGCTTTGCC |
| Primers for mouse *Ccl5* Reverse | Sangon Biotech | CTGGTGTAGAAATACTCCTTGACG |
| Primers for mouse *Gsdma* Forward | Sangon Biotech | CGGGTCTGTCACGGAGCA |
| Primers for mouse *Gsdma* Reverse | Sangon Biotech | CAGCCCTTGGGGATGGTTA |
| Primers for mouse *Gsdmc* Forward | Sangon Biotech | TTGTCAAGGTTGGAGGCAGTG |
| Primers for mouse *Gsdmc* Reverse | Sangon Biotech | AGTTCAGCCAGTAGCCGTGTT |
| Primers for mouse *Gsdmd* Forward | Sangon Biotech | CTTTATGCTTGAAGGGTGA |
| Primers for mouse *Gsdmd* Rorward | Sangon Biotech | TGCTGCCGCTTACCTCC |
| Primers for mouse *Gsdme* Forward | Sangon Biotech | GCAACGGAGGATGGGACG |
| Primers for mouse *Gsdme* Rorward | Sangon Biotech | GGGCAGGTAACACCGCAAA |
| Primers for mouse *Tnf-α* Forward | Sangon Biotech | TTCTCATTCCTGCTTGTGG |
| Primers for mouse *Tnf-α* Reverse | Sangon Biotech | CACTTGGTGGTTTGCTACG |
| Primers for mouse *Il-1β* Forward | Sangon Biotech | CAGGCTCCGAGATGAAC |
| Primers for mouse *Il-1β* Reverse | Sangon Biotech | TGCTTGTGAGGTGCTGA |
| Primers for mouse *Il-6* Forward | Sangon Biotech | TGGGACTGATGCTGGTG |
| Primers for mouse | Sangon Biotech | CTGGCTTTGTCTTTCTTGTTA |

| | | |
|---|---|---|
| *Il-6* Reverse | | |
| Primers for mouse *Il-18* Forward | Sangon Biotech | TTATTGACAACACGCTTTAC |
| Primers for mouse *Il-18* Reverse | Sangon Biotech | TCTGATTCCAGGTCTCC |
| Primers for mouse *Il-10* Forward | Sangon Biotech | TGGACAACATACTGCTAACCGAC |
| Primers for mouse *Il-10* Reverse | Sangon Biotech | CCTGGGGCATCACTTCTACC |
| Primers for mouse *Isg15* Forward | Sangon Biotech | GACGCAGACTGTAGACACGCTT |
| Primers for mouse *Isg15* Reverse | Sangon Biotech | GGGCTTTAGGCCATACTCCC |
| Primers for mouse *Usp18* Forward | Sangon Biotech | GCTGTGGGGAGAAGACGC |
| Primers for mouse *Usp18* Reverse | Sangon Biotech | TGGGCAATCACGGCAAA |
| Primers for mouse *Fis1* Forward | Sangon Biotech | AAGAGGAACAGCGGGACTATG |
| Primers for mouse *Fis1* Reverse | Sangon Biotech | GGATTTGGACTTGGAGACAGC |
| Primers for mouse *Opa1* Forward | Sangon Biotech | AGAATCGGACCCAAGAGCAG |
| Primers for mouse *Opa1* Reverse | Sangon Biotech | TTCGCCAAAACAGGACCAC |
| Primers for mouse *Tfam* Forward | Sangon Biotech | GCATCCCCTCGTCTATCAGTC |
| Primers for mouse *Tfam* Reverse | Sangon Biotech | CCTCCTTCTCCATACCCATCA |
| Primers for mouse *Nrf1* Forward | Sangon Biotech | ACGGAGTGACCCAAACTGAAC |
| Primers for mouse *Nrf1* Reverse | Sangon Biotech | CGCCAAACACCTTGAAGACA |
| Primers for mouse *Pgc1a* Forward | Sangon Biotech | TTTCTGGGTGGATTGAAGTGG |
| Primers for mouse *Pgc1a* Reverse | Sangon Biotech | TCTTTGTGGCTTTTGCTGTTG |
| Primers for mouse *Gapdh* Forward | Sangon Biotech | CCCATCACCATCTTCCAGGAG |
| Primers for mouse *Gapdh* Reverse | Sangon Biotech | TTCACCACCTTCTTCTTGATGTCAT |
| **Plasmids and siRNA** | | |
| pcDNA3.1 | Thermo fisher scientific | V79520 |

| | | |
|---|---|---|
| pcDNA3.1-Flag-tagged mouse ISG15 | This paper | N/A |
| pcDNA3.1-Flag-tagged GSDME | This paper | N/A |
| pcDNA3.1-myc-tagged CPS1 | This paper | N/A |
| siRNAs pool targeting mouse ISG15 | Sangon Biotech | #1 F:GCUGCAAACUCCAUGUUAUTT R:AUAACAUGGAGUUUGCAGCTT #2 F:GGAGCUGUUUGUGAAACAATT R:UUGUUUCACAAACAGCUCCTT #3 F:GCCGUCAAGAGAACAGUUATT R:UAACUGUUCUCUUGACGGCTT |
| **Software and Algorithms** | | |
| GraphPad Prism version 8 | GraphPad software | https://www.graphpad.com/, RRID:SCR_002798 |
| FlowJo v.10 | FlowJo, LLC | https://www.flowjo.com/solutions/flowjo, RRID:SCR_008520 |
| ImageJ | National Institutes of Health | https://imagej.nih.gov/ij/, RRID:SCR_003070 |
| FV10-ASW Viewer software | Olympus | https://www.olympus-lifescience.com.cn, RRID:SCR_014215 |
| PEAKS Studio version X+ | Bioinformatics Solutions Inc. | https://www.bioinfor.com/peaks-studio-x-plus/ |
